# Ni-Decorated B_12_N_12_ Nanocages as High-Performance Platforms for Reversible NH_3_ Sensing: A Comprehensive DFT Study

**DOI:** 10.1021/acs.langmuir.6c02176

**Published:** 2026-09-07

**Authors:** Natanael de Sousa Sousa

**Affiliations:** 37892 Universidade Federal do Maranhão, São Luís, Maranhão 65080-805, Brasil

## Abstract

Selective and reliable NH_3_ detection under realistic environmental conditions remains a major challenge for next-generation chemiresistive gas sensors. Herein, density functional theory (DFT) calculations were employed to systematically investigate six Ni-decorated B_12_N_12_ nanocage configurations to identify the optimal active site for ammonia sensing. Among the investigated systems, Ni@b_66_ exhibited the best sensing performance, combining moderate adsorption strength (*E*_ads_ = −0.82 eV), pronounced band-gap modulation (Δgap = 42.8%), and rapid recovery (τ = 3.74 s at 298 K). PDOS, ELF, CDD, and CDA analyses revealed a donor–acceptor charge-transfer mechanism responsible for the observed electronic and optical responses. Unlike conventional theoretical studies, the sensing mechanism was further validated under realistic conditions through explicit-water molecular dynamics simulations, structural analyses between 173 and 373 K, competitive NH_3_/H_2_O/O_2_ adsorption, and multicomponent atmospheres containing O_2_, N_2_, CO_2_, H_2_O, and CH_4_. The negligible influence of atmospheric O_2_, despite its abundance and reactivity, highlights the robustness and selectivity of the proposed sensor. Benchmark calculations further confirmed the reliability of the computational methodology. These findings establish Ni@b_66_ as a promising boron nitride-based NH_3_ sensor and provide practical design principles for realistic gas-sensing applications.

## Graphical abstract

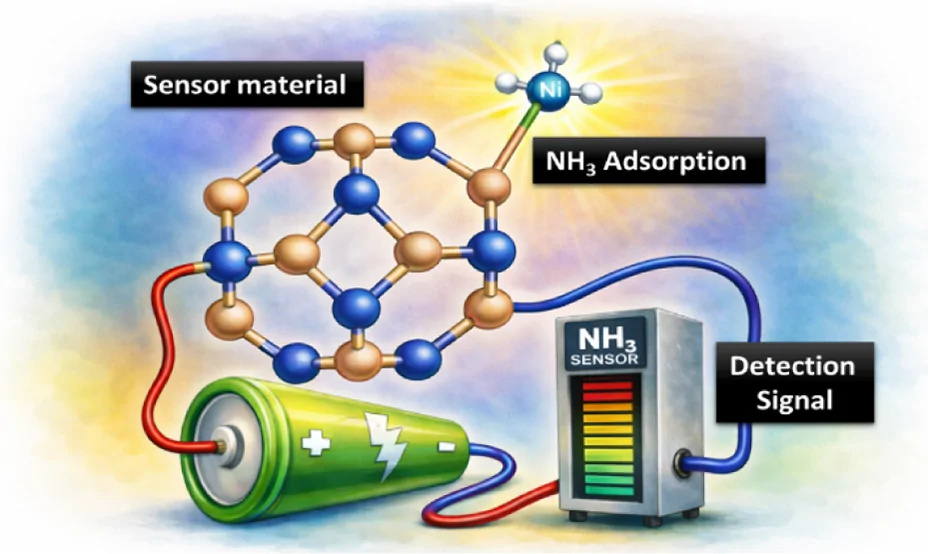

## Introduction

Ammonia (NH_3_) is a toxic and corrosive gas extensively employed in industrial, agricultural, and biomedical processes, whose excessive inhalation may cause severe respiratory and neurological damage.^1–3^ The need to detect NH_3_ at trace concentrations has driven the development of highly sensitive and selective sensors for environmental monitoring, occupational safety, and clinical diagnostics.^1,4,5^ Recent reviews highlight significant advances in inorganic and hybrid materials for NH_3_ detection, emphasizing MEMS-based platforms, metal oxides, and two-dimensional nanomaterials.^1–3,5,6^

Despite advances in resistive, optical, and electrochemical NH_3_ sensors, performance remains limited by the trade-off among sensitivity, selectivity, and room-temperature operation.^2,5^ Conventional solid-state devices often require high temperatures or exhibit slow recovery and cross-sensitivity. Recent strategies employing functional nanomaterials and metal-modified surfaces improve charge transfer and adsorption specificity,^6^ yet challenges persist. Carbon-based systems, for instance, offer tunable properties but limited selectivity.^3^ Studies indicate that coupling conductive matrixes with transition metals (TMs) enhances sensitivity and recovery characteristics.

Recent experimental advances further support this trend. Kim et al.^7^ developed NiO-functionalized graphene micropatterns capable of detecting NH_3_ at concentrations as low as 2.547 ppt at room temperature. Bak et al.^8^ reported CuO/AlN surface acoustic wave sensors operating at 26 °C with response times below 3 s. Hjiri et al.^9^ investigated pure and Ca-doped ZnO films, achieving *R*_n_/*R*_g_ = 33 for 4000 ppm of NH_3_ at 300 °C. Van Duy et al.^10^ demonstrated room-temperature operation under visible light using porphyrin-modified V_2_O_5_, while Seekaew et al.^11^ achieved sub-ppm detection with Ti_3_C_2_T_*x*_-MXene/metal oxide hybrid films. Hao et al.^12^ synthesized Ag–NiFe_2_O_4_ composites exhibiting stable chemiresistive behavior for amine detection at 280 °C, and Aleksanyan et al.^13^ reported enhanced NH_3_ sensitivity in MWCNT-based FET devices.

These findings underscore the potential of TM-containing hybrid systems (Cu, Ni, Fe, Zn) for NH_3_ detection, although room-temperature operation and long-term stability remain strongly dependent on the electronic characteristics of the selected metal.^4,5,7^ Nickel (Ni), in particular, stands out due to its high d-state density and reversible redox behavior, which facilitate charge transfer during the adsorption of electron-donor molecules such as NH_3_.^12^ Integrating the catalytic character of Ni with a chemically stable and insulating matrix such as B_12_N_12_ creates a polarizable interface that promotes selective chemisorption and energy gap modulation, critical factors governing sensitivity and signal reproducibility.^12,14^

The B_12_N_12_ nanocage is a fullerene-like cluster composed of 12 boron and 12 nitrogen atoms arranged in a truncated octahedral geometry with eight hexagonal and six tetragonal rings.^15,16^ It exhibits high thermal stability, a low dielectric constant, and remarkable oxidation resistance, making it suitable for sensing applications under harsh conditions. Since the pioneering syntheses by Golberg et al. and Oku et al.,^15,16^ BN nanocages have been investigated as catalytic supports, storage materials, and sensing platforms. Experimental studies have demonstrated successful encapsulation of transition metals (Fe, Y, Ag, La) within BN nanocapsules, confirming their synthetic feasibility and structural stability.^16^

From a theoretical perspective, TM-modified B_12_N_12_ systems have been extensively explored.^14,17–20^ Previous studies^17^ demonstrated that doping with TMs (Sc–Zn) tunes Δ*E*_gap_ and enhances sensitivity toward polar gases. Ammar et al.^18^ reported that Mn and Fe incorporation modifies the density of states near the Fermi level, favoring NH_3_ chemisorption. Silva and Varela Jr.^19^ observed an adsorption energy of −0.81 eV for Cu@B_12_N_12_, accompanied by significant charge transfer. Nair et al.^20^ correlated chemical hardness and molecular electrostatic potential with adsorption affinity, while Wang et al.^21^ showed that Ni@B_12_N_12_ exhibits Δ*E*_gap_ reduction exceeding 0.4 eV and chemisorptive behavior toward NO_2_, suggesting analogous potential for NH_3_ detection. Ejiofor et al.^22^ compared NH_2_ adsorption on Cu-, Ni-, and Zn-containing nanocages, concluding that Ni-based systems display superior electronic stability and lower activation barriers.

Despite these advances, systematic investigations of NH_3_ adsorption on Ni-decorated B_12_N_12_ nanocages remain scarce, particularly regarding the influence of specific active sites (e.g., b_64_ vs b_66_) on sensing performance. The relationship among the adsorption strength, electronic modulation, and reversibility remains incompletely understood. In this context, this work employs DFT combined with multilevel analyses to elucidate the site-dependent adsorption mechanisms and identify the key parameters governing the sensitivity and reversibility in Ni-decorated B_12_N_12_-based NH_3_ sensors.

## Computational Methodology

All calculations were performed within the framework of density functional theory (DFT) using the ORCA 5.0 package.^23^ Geometry optimizations were carried out employing the composite B97-3c functional proposed by Grimme and coworkers,^24^ which combines the B97 generalized gradient approximation with the D3­(BJ) dispersion correction and the geometrical counterpoise (gCP) correction, providing an accurate and computationally efficient description of structural and energetic properties of TM-modified systems. The 6-31G­(d,p) basis set was employed to adequately describe the valence and polarization effects of B, N, and Ni atoms.^25–27^ All structures were fully optimized without symmetry constraints, and harmonic frequency calculations confirmed the absence of imaginary frequencies, ensuring that all stationary points correspond to true minima on the potential energy surface. Tight convergence thresholds were adopted, including an energy change of 5 × 10^–6^ Hartree, an RMS gradient of 1 × 10^–4^ Hartree Bohr^–1^, and a maximum displacement of 4 × 10^–3^ Bohr. Tight convergence criteria were adopted to ensure reliable geometries: energy of 5 × 10^–6^ Hartree, RMS gradient of 1 × 10^–4^ Hartree/Bohr, and maximum displacement of 4 × 10^–3^ Bohr, ensuring reliable equilibrium geometries for all investigated systems.^28^

The cohesive energy (*E*_coh_) was calculated as the difference between the total energy of the optimized nanocage and the sum of the energies of its constituent isolated atoms (B, N, and Ni) and then normalized by the total number of atoms in the system.^29^ In addition, the global reactivity descriptors, including the chemical potential (μ), chemical hardness (η), and electrophilicity index (ω), were calculated from the frontier molecular orbital energies to evaluate the structural stability and electronic reactivity of the investigated systems (eqs 1–3).^30,31^

1 $$\eta \approx \frac{1}{2}\left(E_{\mathrm{LUMO}}-E_{\mathrm{HOMO}}\right)$$

2 $$\mu \approx \frac{1}{2}\left(E_{\mathrm{LUMO}}+E_{\mathrm{HOMO}}\right)$$

3 $$\omega =\frac{\mu^{2}}{2\eta }$$

The systems stability study uses cohesive energy and also uses Gibbs free energy (Δ*G*_*ads*_) and enthalpy analysis (Δ*H*_ads_) which are calculated according to eqs 4 and 5:

4 $$\Delta G_{\mathrm{ads}}=G_{\left(\mathrm{nanocage}-{\mathrm{NH}}_{3}\right)}-\left(G_{\left(\mathrm{nanocage}\right)}+G_{\left({\mathrm{NH}}_{3}\right)}\right)$$

5 $$\Delta H_{\mathrm{ads}}=H_{\left(\mathrm{nanocage}-{\mathrm{NH}}_{3}\right)}-\left(H_{\left(\mathrm{nanocage}\right)}+H_{\left({\mathrm{NH}}_{3}\right)}\right)$$

Where, $G/H_{\left(\mathrm{nanocage}-{\mathrm{NH}}_{3}\right)}$ is the free energy or enthalpy of the system after adsorption of NH_3_, *G*/*H*_(nanocage)_ is the free energy or enthalpy of the isolated cage, and $G/H_{\left({\mathrm{NH}}_{3}\right)}$ is the Gibbs free energy or enthalpy of NH_3_.

The energy gap (*E*_gap_ = *E*_LUMO_ – *E*_HOMO_) was used as the primary metric of the sensor’s electronic sensitivity, indicating changes in nanocage conductivity after NH_3_ adsorption. The adsorption energy (*E*_ads_) was calculated as the difference between the total energy of the complex (*E*_nanocage/NH_3__) and the sum of the energies of the isolated nanocage and NH_3_ molecule, including the basis set superposition error (BSSE) (*E*_nanocage_ – *E*_NH_3__ + BSSE). Negative *E*_ads_ values indicate spontaneous adsorption. The BSSE was calculated as proposed in the ORCA documentation (see eq S1 in the Supporting Information). Charge transfer (*Q*_t_) between the gas and the nanomaterial was evaluated using Mulliken population analysis.^29^

For work function sensor applications,^32^ the variation in the sensor’s electrical current density (*j*) can be experimentally correlated with the work function (Φ) before and after the interaction of the nanocage with the molecule to be detected, according to eq 6:

6 $$j=AT^{2}e^{\left(\frac{-\Phi }{k_{B}T}\right)}$$

where *A* is the Richardson–Dushman constant (theoretical value 120.1 A m^–2^ K^–2^),^49^ *T* is the thermodynamic temperature (K), and *k*_B_ is the Boltzmann constant (8.62 × 10^–5^ eV K^–1^). Sensor efficiency was also evaluated through the recovery time (τ),^33^ which is related to the adsorption energy via the Arrhenius equation (eq 7):

7 $$\tau =\nu_{0}^{-1}e^{-E_{\mathrm{ads}}/k_{B}T}$$

where ν = 5.2 × 10^14^ s^–1^ represents the attempt frequency, which corresponds to visible light frequency.^17,28,34,35^ Lower *E*_ads_ values correspond to shorter recovery times, indicating higher efficiency for reversible sensor applications. The electronic structures were further analyzed through total and partial density of states (TDOS and PDOS) using the Multiwfn 3.7 package.^36^ The energy scale was referenced to the corresponding Fermi level (*E*_F_ = 0 eV) to facilitate comparison of adsorption-induced electronic changes. Charge density difference (CDD) maps were generated by subtracting the electron densities of the isolated fragments (cage and NH_3_) from that of the adsorption complex while maintaining the fragment geometries fixed at the optimized complex configuration. Charge decomposition analysis (CDA) was performed by considering the nanocage and NH_3_ as independent fragments, allowing the decomposition of the interaction into charge donation, back-donation, and polarization contributions. The nature of the adsorption interaction was further investigated using the electron localization function (ELF), the reduced density gradient (RDG), and the noncovalent interaction (NCI) analyses, all performed with Multiwfn 3.7. RDG isosurfaces were colored according to the sign­(λ_2_)­ρ descriptor to distinguish attractive, dispersive, and repulsive interactions.^37,38^

Optical properties were investigated using time-dependent density functional theory (TD-DFT). Using the same level of theory, 100 excited states were calculated to simulate the UV–vis absorption spectra, and the dominant orbital contributions associated with each electronic transition were analyzed using oscillator strengths and molecular orbital compositions.^17,39^ Finally, molecular dynamics (MD) simulations were performed at the GFN1-xTB level using the xTB MD module under the canonical (NVT) ensemble. Simulations were carried out at 298.15 K for 1000 ps using a 2 fs integration step to analyze the dynamic stability of the systems. Additional simulations employing an explicit water box containing 60 H_2_O molecules and temperature-ramp protocols (173–373 K) were performed to evaluate adsorption stability under humid and variable-temperature conditions.

## Results and Discussion

### Structural Analysis

The B_12_N_12_ nanocage exhibits high symmetry and is composed of 12 boron (B) and 12 nitrogen (N) atoms distributed over equivalent vertices of a polyhedral framework. This structure is decorated with a nickel (Ni) atom in six distinct initial configurations,^27,40^ as illustrated in Figure 1:

- Ni@b_64_—Ni placed on the B–N bond connecting a hexagonal and tetragonal ring (b_64_).
- Ni@b_66_—Ni located above the B–N bond shared by two adjacent hexagonal rings (b_66_).
- Ni@(N)—Ni placed directly above a nitrogen atom.
- Ni@(B)—Ni positioned directly above a boron atom.
- Ni@(r4)—Ni centered above a tetragonal ring (r4 site).
- Ni@(r6)—Ni centered above a hexagonal ring (r6 site).

**Figure 1:**
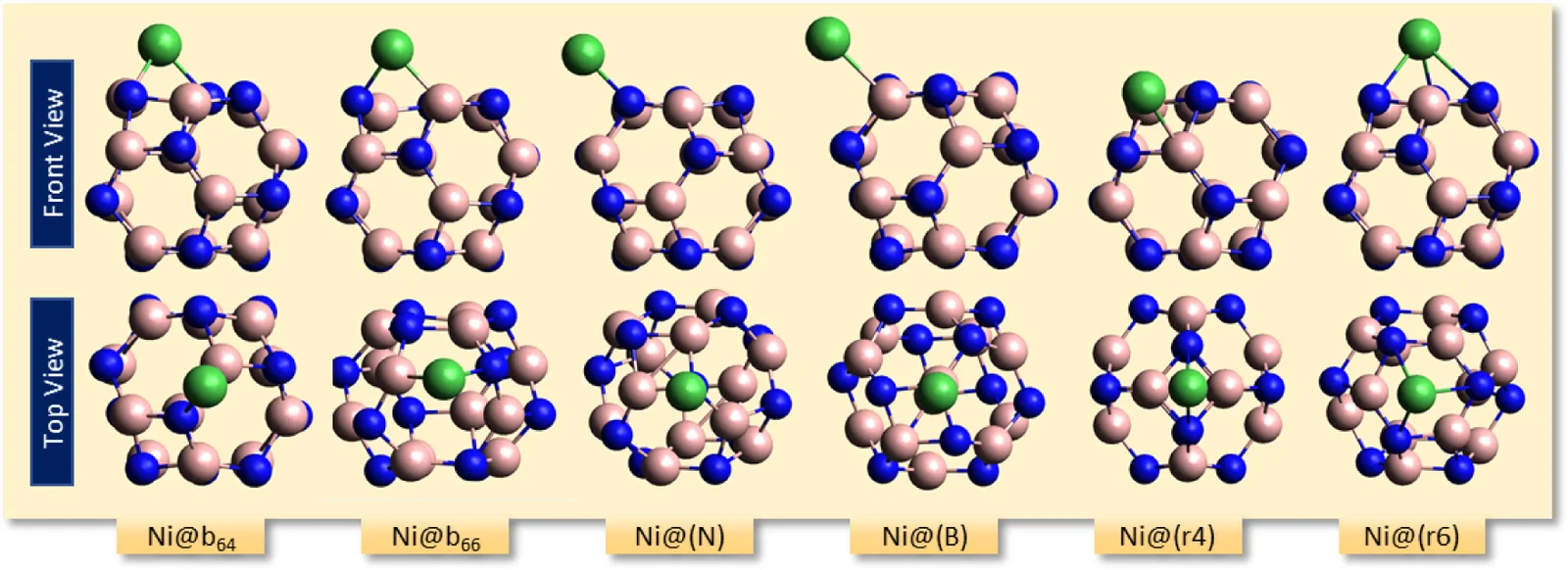
All configurations of the Ni-decorated B_12_N_12_ nanocages tested (over b_64_ and b_66_ bonds (Ni@b_64_ and Ni@b_66_), top on B and N atoms (Ni@(B) and Ni@(N) and over the rings r4 and r6 (Ni@(r4) and Ni@(r6). The B atom is shown in pink color, the N atom in blue, and the Ni atom in green.

These configurations were selected to systematically evaluate the influence of distinct adsorption sites, vertex, bridge, and ring-centered positions, on the structural stability and electronic properties of the Ni-decorated nanocages.

These configurations underwent full-geometry relaxation. The total energies (Eh) and final geometries are presented in Table 1 and Figure 2. The results indicate that Ni@b_64_ exhibits the lowest total energy, establishing it as the thermodynamically most stable structure. It is observed that geometries initially positioned at Ni@(N) and Ni@(B) converge to bridge configurations (b_66_ and b_64_, respectively), suggesting that these bridge sites are energetically more favorable than positions directly above individual atoms.^41^ The Ni@b_66_ configuration also remains stable after optimization, although slightly less stable than Ni@b_64_, whereas Ni@(r4) only partially preserves its initial topology. These results confirm the preference of Ni for B–N bridge sites, with b_64_ and b_66_ representing the most stable modes, in agreement with previous reports.^27^

**Table 1:** Initial Geometry Tested, Total Energy, and New Configuration after Optimization

Initial Geometry	After Optimization	Energy (Eh)
Ni@b_64_	Ni@b_64_	–2463.8595
Ni@b_66_	Ni@b_66_	–2463.8126
Ni@(N)	Ni@b_66_	–2463.7560
Ni@(B)	Ni@b_64_	–2463.8017
Ni@(r4)	Ni@(r4)	–2463.7976
Ni@(r6)	Ni@b_66_	–2463.8189

**Figure 2:**
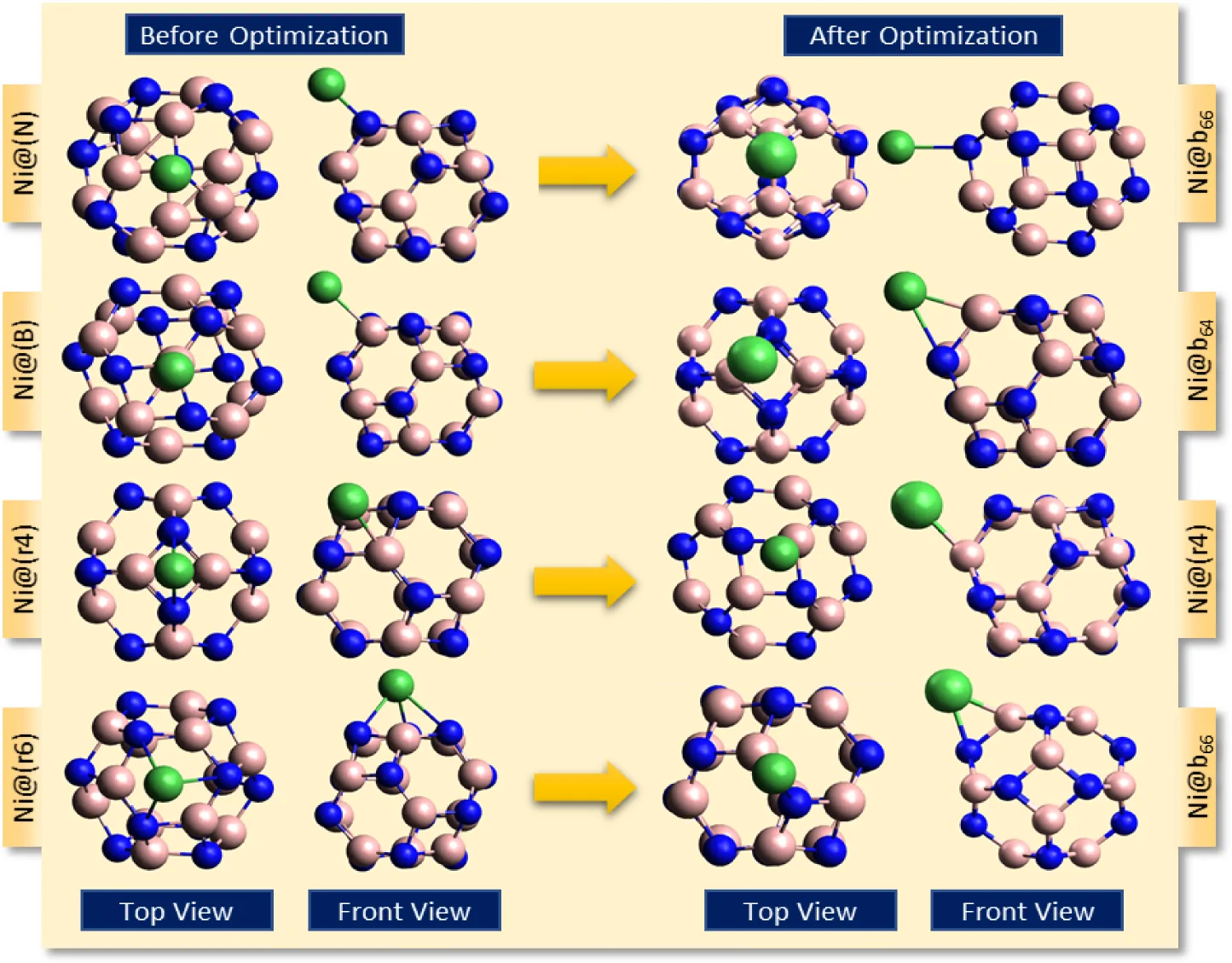
Changes in the nanocage configurations after geometry relaxation. The B atom is shown in pink, the N atom in blue, and the Ni atom in green.

Different spin multiplicities of the Ni-decorated B_12_N_12_ nanocages (i.e., singlet and triplet) were evaluated to determine the energy and the most stable configuration of each structure, as shown in Table 2. It can be seen that the B_12_N_12_ nanocage has a singlet spin; the Ni metal is more stable with a singlet spin state in the b_64_ system and a triplet state in the b_66_ system, as observed in previous work. This finding agrees with results published by Arshad et al.^42^ The spin contamination of the Ni-decorated B_12_N_12_ nanocage was also analyzed. Table 2 summarizes the values obtained for the total spin operator expectation value ⟨*Ŝ* ^2^⟩, their theoretical expectations, and the deviations. The B_12_N_12_ and Ni@b_64_ systems showed no deviations, indicating that the spin states are well described, while Ni@b_66_ showed a small deviation (0.0082) with negligible contamination. This supports the stability of the spin states and confirms that the chosen functional/basis set combination is appropriate for these systems.

**Table 2:** Relative Energy (Eh) of Ni Atom Spin States and Spin-Contamination Analysis

System	Singlet	Triplet	⟨*Ŝ*^2^⟩	⟨*Ŝ*^2^⟩ Expected	Deviation
B_12_N_12_	0	-	0.000000	0.000000	0.000000
Ni@b_64_	0	0.19	0.000000	0.000000	0.000000
Ni@b_66_	0.22	0	2.008265	2.000000	0.008265

The most stable optimized structures in Figure 3 reveal that the pristine B_12_N_12_ nanocage preserves its high Th symmetry and a null dipole moment (0.00 D), with average B–N bond lengths ranging from 1.43 to 1.48 Å, confirming its intrinsic structural robustness. Upon Ni decoration at the b_64_ and b_66_ sites, symmetry breaking occurs, accompanied by a significant increase in dipole moment, reaching 3.31 D for Ni@b_64_ and 2.10 D for Ni@b_66_. In the Ni@b_64_ configuration, Ni–N/B interaction distances of 1.74–1.80 Å indicate strong coordination and pronounced electronic polarization. In contrast, Ni@b_66_ exhibits slightly longer Ni–N/B distances (1.91–1.95 Å), suggesting comparatively weaker interaction strength. These structural features are consistent with the higher thermodynamic stability of Ni@b_64_ and demonstrate that bridge-site coordination enhances electronic redistribution within the nanocage framework.

**Figure 3:**
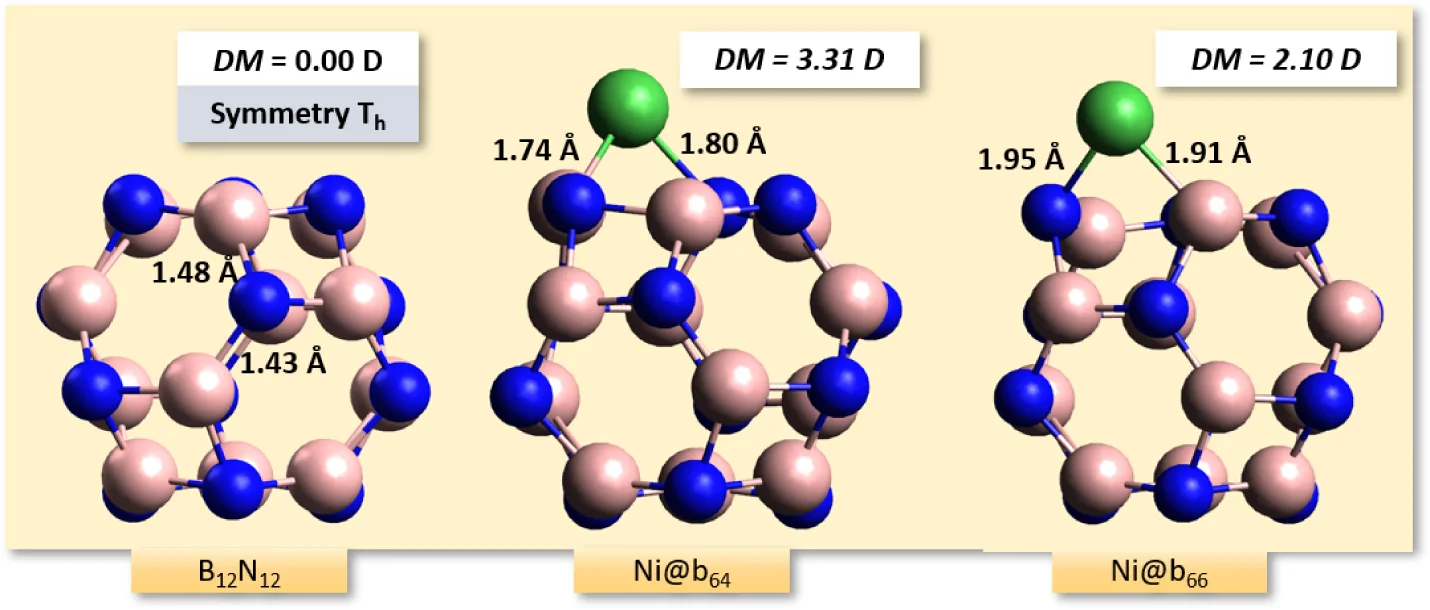
Dipole moments and optimized structures of B_12_N_12_, Ni@b_64_ and Ni@b_66_ nanocages. The B atom is shown in pink, the N atom in blue, and the Ni atom in green.

After relaxation of the isolated nanocages and identification of the most stable decorated structures, the free adsorption of the ammonia molecule was subsequently investigated (Figure S2 and Table S4 in the Supporting Information). Figure 4 shows that NH_3_ adsorption occurs preferentially through coordination of the nitrogen lone pair to the metallic center, characterizing a Lewis acid–base interaction, as predicted by Rostamoghli et al.^43^ and Ammar et al.^18^ For the pristine nanocage (B_12_N_12_–NH_3_), an N–B distance of 1.63 Å and a dipole moment of 7.98 D are observed, indicating moderate interaction strength. In the Ni@b_64_–NH_3_ and Ni@b_66_–NH_3_ systems, the Ni–N distances of 1.89 and 1.98 Å, respectively, indicate a less pronounced interaction, accompanied by an increase in dipole moment (8.22 and 9.59 D), reflecting enhanced charge separation within the structures. The decrease in bond order (BO ≈ 0.50 → 0.46) and the vibrational red shift (1338 → 1254 cm^–1^) suggest significant electronic redistribution and are consistent with the observed bond elongation. These findings are consistent with previous studies reporting the strong affinity of NH_3_ toward metallic sites in doped nanomaterials due to d–lone-pair interactions.^44,45^

**Figure 4:**
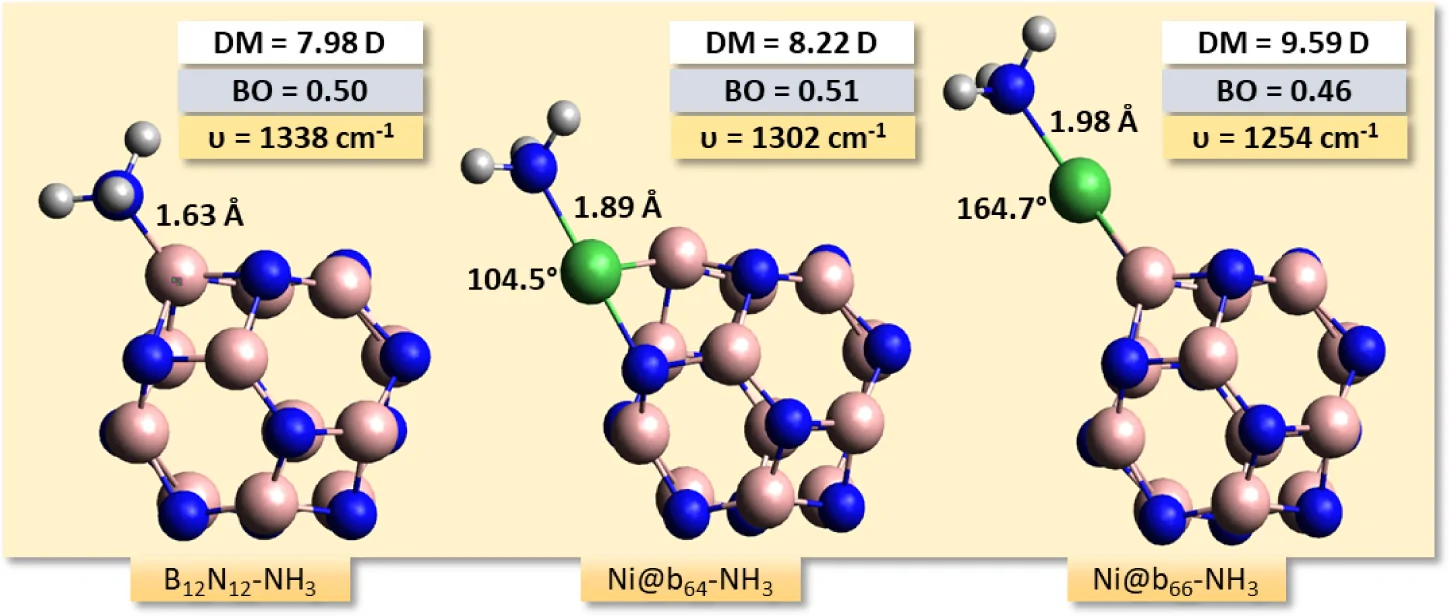
Dipole moment, cage-NH_3_ bond order, and bond frequency of optimized geometries of nanocages with an adsorbed NH_3_ molecule. The B atom is shown in pink, the N atom in blue, the Ni atom in green, and the H atom in white.

To validate the functional/basis set used in this work, a benchmark test was performed with variation of the basis set (Figure S1a) and strategic variation of the functional (Figure S1b in the Supporting Information) for the B_12_N_12_ and Ni@B_12_N_12_ systems after ammonia adsorption. The results show that the application of more robust basis sets, such as triple-ζ, did not produce a significant variation in the total energy of the systems, but LANL2DZ, with a pseudopotential for Ni, presented lower energy than the other basis sets. The use of functionals such as B3LYP with D3 dispersion, HSE06, M06, or the MP2 method did not produce significantly different results from those of B97-3c. Given this consistency, the B97-3c/6-31G­(d,p) level offers an ideal balance between computational efficiency and reliable energy descriptions, making it suitable for the systematic screening of adsorption and detection properties. To provide a quantitative assessment of the adopted computational protocol, the relative deviations of the tested functionals and basis sets were calculated with respect to the B97-3c/6-31G­(d,p) methodology employed throughout this work. As shown in Table X, the calculated deviations remain below 0.13% for all comparable methods, indicating excellent agreement and confirming the reliability and consistency of the selected computational approach. The LANL2DZ basis set was not included in the quantitative error analysis because it employs an effective core potential (ECP) for Ni, resulting in total electronic energies that are not directly comparable to the all-electron basis sets used in the remaining benchmark calculations.

### Electronic Analysis

Ni decoration markedly enhances the electronic sensitivity of the B_12_N_12_ nanocage by reducing the band gap and introducing metal-derived states near the Fermi level. As summarized in Table 3, pristine B_12_N_12_ exhibits a minimal response upon NH_3_ adsorption (Δ*E*_gap_ = 1.51%), confirming its limited sensing capability. In contrast, Ni@b_64_ shows a pronounced gap increase (Δ*E*_gap_ = 80.67%), indicating strong electronic perturbation but potentially limited reversibility. The Ni@b_66_ system exhibits a more balanced behavior, with moderate gap variation in the α state (2.40%) and significant modulation in the β state (42.85%), associated with its initially narrow band gap.

**Table 3:** HOMO Energy (*E*_H_), LUMO Energy (*E*_L_), HOMO-LUMO Gap (*E*_gap_), Variation of the Gap (Δ*E*_gap_), and Electronic Sensitivity in Percentage (Δgap) Values, for Isolated and NH_3_ Adsorbed Systems

		Isolate System			NH_3_ Adsorbed				
System	S	*E*_H_ (eV)	*E*_L_ (eV)	*E*_gap_ (eV)	*E*_H_ (eV)	*E*_L_ (eV)	*E*_gap_ (eV)	Δ*E*_gap_ (eV)	Δgap (%)
B_12_N_12_	α	–6.72	–1.44	5.29	–5.83	–0.62	5.21	–0.08	1.51
Ni@b_64_	α	–4.41	–3.22	1.19	–3.78	–1.63	2.15	0.96	80.67
Ni@b_66_	α	–4.17	–2.09	2.08	–3.19	–1.15	2.03	–0.05	2.40
	β	–5.22	–4.94	0.28	–4.57	–4.17	0.40	0.12	42.85

In chemoresistive sensors, electrical conductivity (σ) is a key parameter, since sensor response depends on conductivity changes during molecule-nanocage interaction. Conductivity can be expressed as $\sigma =AT^{3/2}e^{\left(-E_{\mathrm{gap}}/2k_{B}T\right)}$ ,^46^ where *A* is a constant, *T* is the temperature, and *k*_B_ is the Boltzmann constant. Therefore, small variations in *E*_gap_ lead to exponential changes in σ. Consequently, systems exhibiting larger Δgap, such as Ni@b_64_, are expected to generate stronger electrical signals. This balance between electronic response and moderate interaction strength highlights Ni@b_66_ as the most suitable configuration for reversible NH_3_ sensing, and the band gap variations fall within experimentally reported ranges for chemiresistive NH_3_ sensors based on nanostructured materials, supporting the practical relevance of the predicted response. Although this equation was originally derived for bulk semiconductors, its application in theoretical studies of nanoscale sensors has been widely adopted as a qualitative descriptor of the electronic response induced by adsorption.^47–50^ In the present work, this expression was not employed to predict the absolute electrical conductivity of the nanocages, but rather to compare the relative conductivity changes among the investigated systems, assuming that variations in the HOMO-LUMO energy gap reflect changes in the ease of electronic excitation and, consequently, in the chemiresistive response of the sensing material. Therefore, the calculated conductivity values should be interpreted as comparative indicators of electronic sensitivity rather than quantitative predictions of experimentally measurable conductivity.

Through TDOS and PDOS diagrams in Figure 5, the evolution of the electronic structure upon Ni decoration and subsequent NH_3_ adsorption is illustrated. For pristine B_12_N_12_, a wide band gap is observed, with the HOMO mainly composed of N 2p orbitals and the LUMO predominantly derived from B 2p orbitals, reflecting the polar nature of B–N bonds and the intrinsic semiconducting character of the cage. The energy values are referenced to their respective Fermi levels, such that 0 eV corresponds to E_F_ in all plots. For isolated Ni@b_64_ and Ni@b_66_ systems, additional states emerge near the Fermi level, primarily associated with Ni 3d orbitals, significantly reducing the band gap. The PDOS indicates strong d-state contributions of Ni metal near the HOMO region, evidencing Ni–N and Ni–B interactions, consistent with the previously discussed structural polarization. After NH_3_ adsorption, noticeable shifts in the density of states near the HOMO and LUMO are observed, along with enhanced overlap between Ni 3d orbitals and the NH_3_ nitrogen lone pair (2p). This hybridization induces electronic reorganization and band gap modulation, particularly pronounced for Ni@b_64_–NH_3_, supporting its high sensitivity and superior predicted chemoresistive sensing performance.

**Figure 5:**
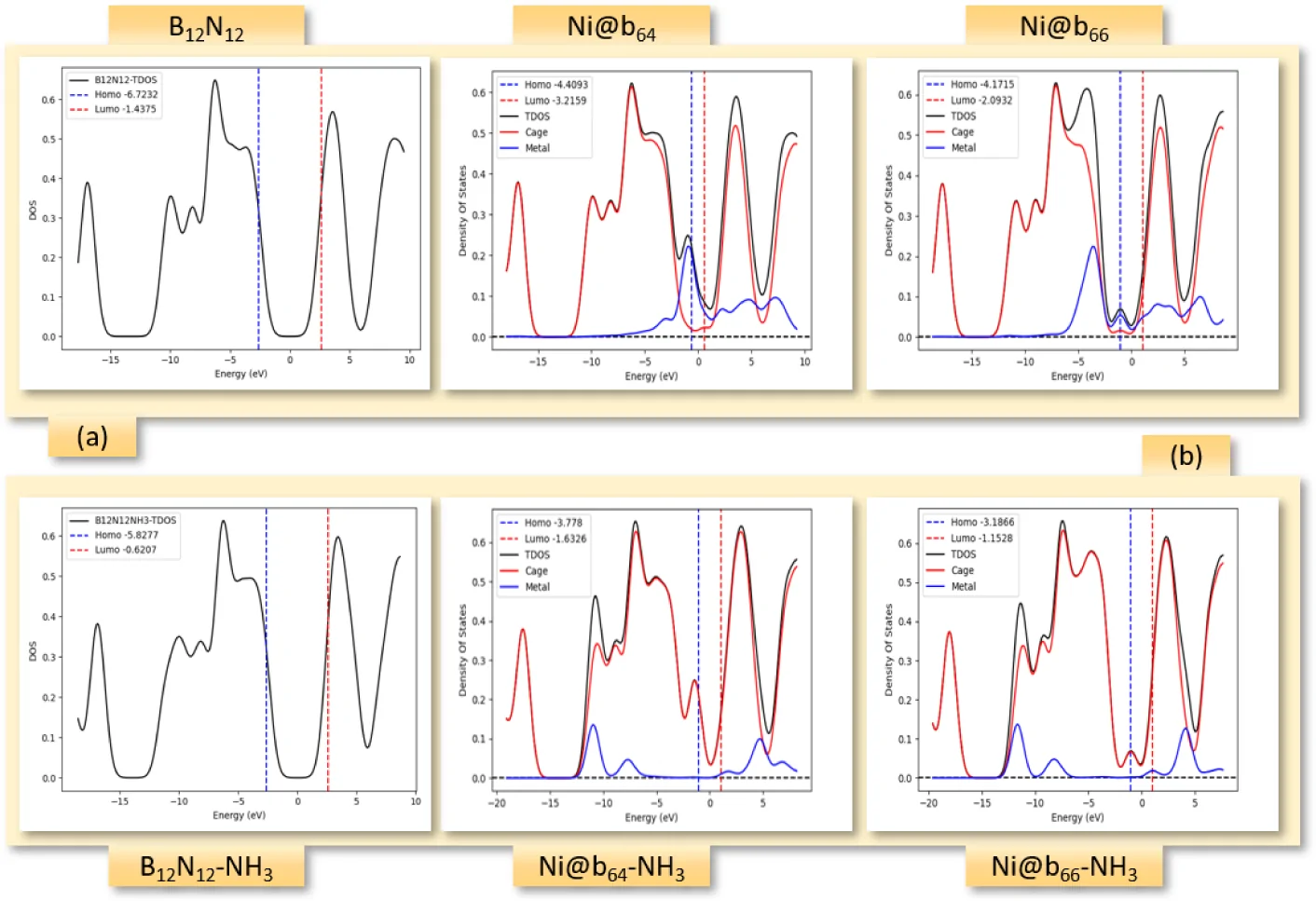
TDOS and PDOS plots of isolated nanocages, B_12_N_12_, Ni@b_64_ and Ni@b_66_ (a) and NH_3_ adsorbed systems B_12_N_12_–NH_3_, Ni@b_64_–NH_3_ and Ni@b_66_–NH_3_ (b).

The frontier molecular orbitals (HOMO and LUMO) of the nanocages before and after NH_3_ adsorption are presented in Figure 6, enabling a correlation between the spatial distribution of electron density and the DOS results. For pristine B_12_N_12_, the HOMO is predominantly localized on nitrogen atoms, whereas the LUMO is mainly distributed over boron atoms, confirming the intrinsic donor–acceptor character and the wide band gap observed in the TDOS. In isolated Ni@b_64_ and Ni@b_66_ systems, the HOMO shows a significant contribution from Ni 3d orbitals, evidencing metal–cage affinity, consistent with the emergence of Ni-derived states near the Fermi level in the PDOS plots. The LUMO also exhibits partial Ni participation, contributing to band gap reduction.

**Figure 6:**
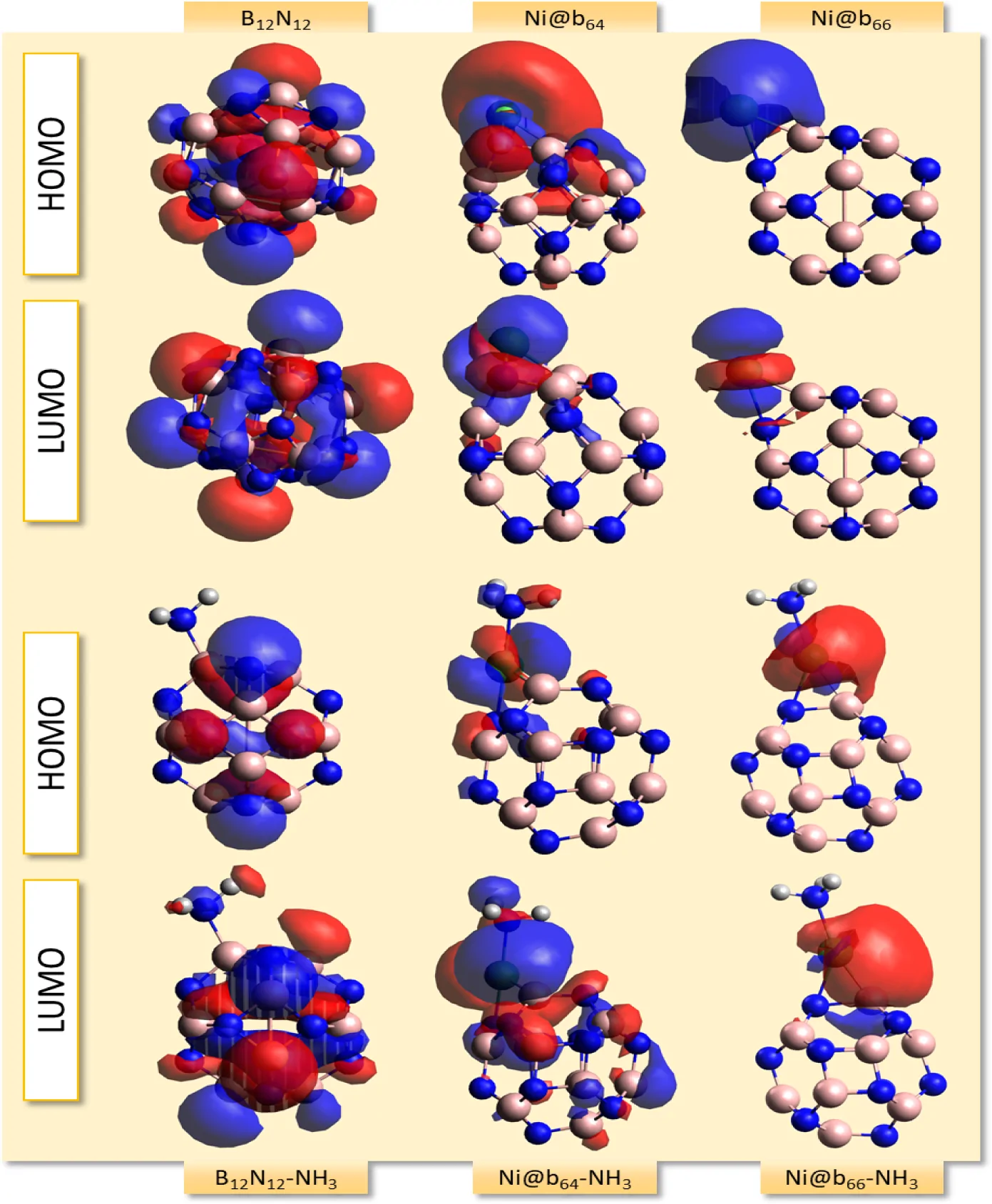
HOMO and LUMO data for the ground-state geometry of B_12_N_12_, Ni@b_64_ and Ni@b_66_ nanocages before and after NH_3_ adsorption.

After NH_3_ adsorption, the HOMO becomes redistributed over both the metallic center and the adsorbed molecule on the Ni@b_64_–NH_3_ complex, while the LUMO shows increased delocalization or partial localization on NH_3_, B_12_N_12_–NH_3_, and Ni@b_64_–NH_3_, indicating charge transfer and d–lone-pair interaction. On the other hand, in the Ni@b_66_–NH_3_ system, the HOMO and LUMO orbitals are predominantly located over the metal and the cage, with a smaller contribution from ammonia. These spatial changes support the observed *E*_gap_ and Δgap variations, particularly pronounced in Ni-modified nanocages, reinforcing their potential for sensing applications.

### Adsorption and Recovery Time Analysis

Figure 7 presents the calculated adsorption energies (*E*_ads_) of NH_3_ on the B_12_N_12_, Ni@b_64_, and Ni@b_66_ nanocages, computed both without and with basis set superposition error (BSSE) correction. The adsorption energy values indicate a chemisorption interaction type, which is characterized by a high adsorption energy (*E*_ads_ < −0.3 eV) as proposed by Peng et al. in their study for NO and C_2_H_2_ detection^51^ and corroborated by Kaewmaraya et al.^52^ applying phosphorene for NH_3_, NO, NO_2_, CO and CO_2_ gases sensing. Without the correction, the adsorption energies are −1.30 eV (B_12_N_12_–NH_3_), −2.06 eV (Ni@b_64_–NH_3_), and −1.36 eV (Ni@b_66_–NH_3_), indicating the highest affinity for the Ni@b_64_ system. After BSSE correction, the values become less negative (−1.28, −1.69, and −0.82 eV, respectively), confirming that basis set overlap artificially overestimates the interaction strength. The BSSE correction led to a significant reduction in adsorption energy due to the use of localized Gaussian basis functions and the short interaction distances between NH_3_ and the Ni sites. In such cases, there is increased artificial sharing of basis functions between the fragments, resulting in an overestimation of the complex’s stability. Applying the counterpoise correction eliminates this numerical artifact, yielding more reliable adsorption energies. This substantial correction is consistent with strongly interacting molecular systems described by intermediate-sized basis sets and ensures the consistency of comparisons across the various systems investigated.

**Figure 7:**
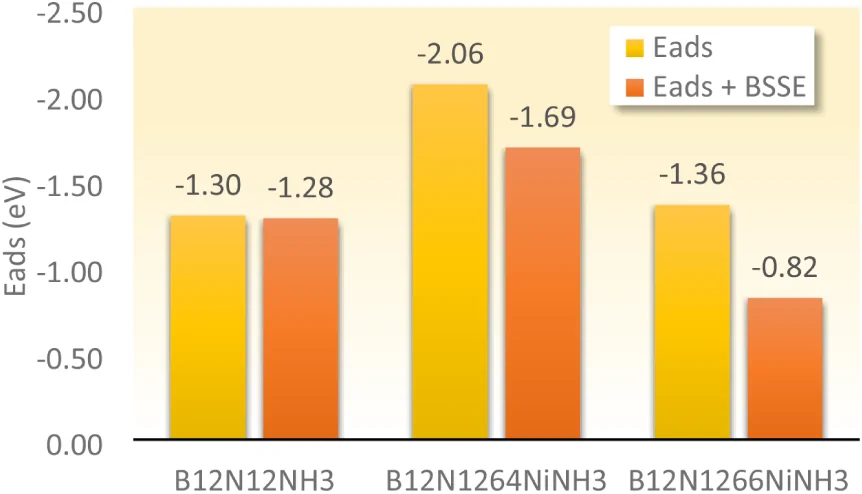
Adsorption energies without and with BSSE correction for B_12_N_12_–NH_3_, Ni@b_64_–NH_3_ and Ni@b_66_–NH_3_ systems.

From a sensing perspective, excessively high adsorption energies may hinder reversibility by increasing the recovery time, whereas moderate values favor rapid response and reusability. Therefore, Ni@b_64_ exhibits high sensitivity but may present slower recovery, while Ni@b_66_ shows a weaker interaction and potentially improved performance in reversible ammonia sensing. The inclusion of BSSE correction was essential to avoid overestimation of chemisorption and to ensure reliable predictions of sensor performance.

Recovery time (τ) analysis is used to estimate the time required for the desorption of the adsorbed molecule from the nanocage, thereby freeing the nanocage surface for a new interaction. The selected attempt frequency (ν = 5.2 × 10^14^ s^–1^) corresponds to the vibrational frequency of visible light (yellow) and is commonly used for nanometric systems that absorb in this region of the spectrum, as is the case with Ni-decorated nanocages, both before and after NH_3_ adsorption, as will be shown later. Figure 8 presents the recovery time, in seconds, for NH_3_ adsorbed in B_12_N_12_, Ni@b_64_, and Ni@b_66_ nanocages as a function of system *E*_ads_ and temperature (250–800 K). An exponential decrease in τ is observed with increasing temperature, in agreement with Arrhenius behavior (eq 4). The Ni@b_64_–NH_3_ system exhibits the longest recovery times across the entire temperature range (>100 h at 300 K), reflecting its more negative adsorption energy (*E*_ads_ = −1.69 eV), which implies slower desorption kinetics. B_12_N_12_–NH_3_ shows intermediate values, but over 100 h as well, whereas Ni@b_66_–NH_3_ exhibits the shortest recovery times (τ = 3.74 s at room temperature), consistent with its lower *E*_ads_ after BSSE correction and with experimentally reported response/recovery dynamics in room-temperature NH_3_ sensors. It is possible to observe that the correlation is direct: the greater the magnitude of *E*_ads_, the higher the desorption energy barrier and, consequently, the longer the recovery time. From a sensing perspective, excessively long recovery times compromise reversibility and cyclic applications; therefore, Ni@b_66_ tends to provide better performance in applications that require fast response and efficient regeneration, while Ni@b_64_ favors greater sensitivity and strong adsorption, characteristics indicated for capture and storage systems, as employed in previous work for CO capture.^53^

**Figure 8:**
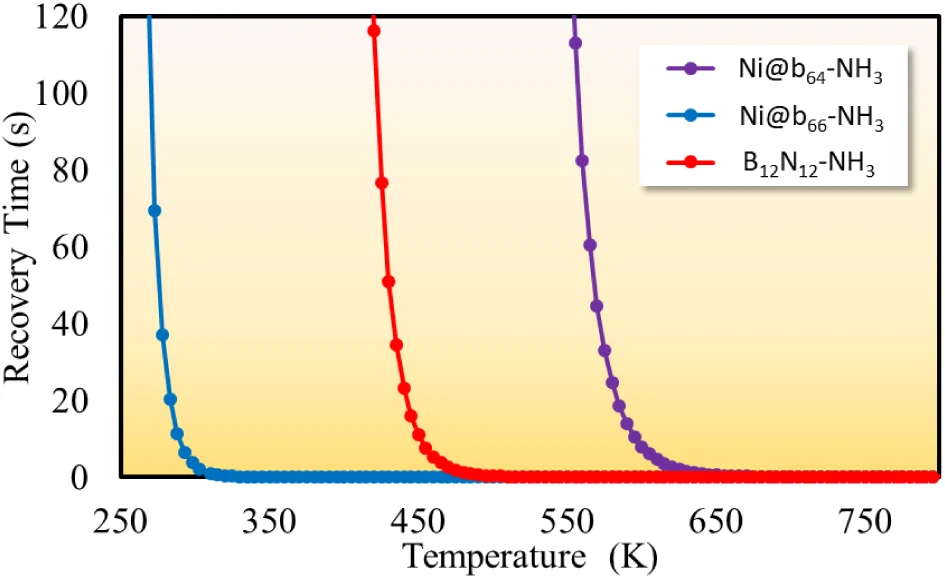
Recovery time (τ) calculated for the B_12_N_12_–NH_3_, Ni@b_64_–NH_3_ and Ni@b_64_–NH_3_ systems as a function of temperature.

### Work Function Analysis

The work function (Φ) represents the minimum energy required to remove an electron from the Fermi level (FL) to the vacuum level and was estimated as Φ = *E*_vac_ – *E*_F_, assuming the vacuum level as the reference (*E*_vac_ = 0 eV). Table 4 summarizes the calculated Fermi levels, work functions, and their variations after NH_3_ adsorption. Pristine B_12_N_12_ exhibits the highest work function (4.08 eV), whereas Ni decoration shifts the Fermi level toward the vacuum level, reducing Φ to 3.81 eV for Ni@b_64_ and 3.13 eV for the α-spin channel of Ni@b_66_. Upon NH_3_ adsorption, all systems exhibit a pronounced decrease in Φ, reaching reductions of 21.08%, 28.87%, and 30.67% for B_12_N_12_, Ni@b_64_, and Ni@b_66_, respectively, indicating that adsorption facilitates electron emission from the sensing material. This behavior is consistent with the charge redistribution revealed by CDD, CDA, ELF, and PDOS analyses shown in the next sections, which demonstrate donor–acceptor interaction between the NH_3_ lone pair and the Ni-derived electronic states. The largest work-function modulation observed for Ni@b_66_ further confirms its superior electronic response and highlights its potential for work-function-based NH_3_ sensing.

**Table 4:** Spin (S), Fermi Level (FL), Work Function (Φ), and Work Function Variation (ΔΦ) Values for the Isolated and NH_3_-Adsorbed Nanocages

Systems	S	FL (eV)	Φ (eV)	ΔΦ (%)
B_12_N_12_	α	–4.08	4.08	
Ni@b_64_	α	–3.81	3.81	
Ni@b_66_	α	–3.13	3.13	
Ni@b_66_	β	–5.08	5.08	
B_12_N_12_–NH_3_	α	–3.22	3.22	21.08
Ni@b_64_–NH_3_	α	–2.71	2.71	28.87
Ni@b_66_–NH_3_	α	–2.17	2.17	30.67
Ni@b_66_–NH_3_	β	–4.37	4.37	13.98

### Stability Analysis

Stability analysis is essential for assessing the structural and thermodynamic feasibility of nanomaterials intended for sensing applications, as it links structural robustness, chemical reactivity, and the spontaneity of the adsorption process. Table 5 compiles global reactivity descriptors, chemical hardness (η), chemical potential (μ), and electrophilicity (ω), together with cohesive energy (*E*_coh_), adsorption enthalpy (Δ*H*_ads_), and Gibbs free energy (Δ*G*_ads_). Those descriptors, grounded in conceptual DFT, are widely used to interpret stability and reactivity trends in nanostructured systems.^54,55^

**Table 5:** Chemical Hardness (η), Chemical Potential (μ), Electrophilicity (ω), Cohesion Energy (*E*_coh_), Adsorption Enthalpy (Δ*H*_ads_) and Gibbs Energy (Δ*G*_ads_) Values for the Isolated and NH_3_-Adsorbed Nanocage Systems

	Isolate System				NH_3_ Adsorbed				
System	η (eV)	μ (eV)	ω (eV)	*E*_coh_ (eV)	η (eV)	μ (eV)	ω (eV)	Δ*H*_ads_ (eV)	Δ*G*_ads_ (eV)
B_12_N_12_	2.64	–4.08	3.15	–7.68	2.6	–3.22	2.00	–1.32	–0.85
Ni@b_64_	0.60	–3.81	12.18	–7.67	1.07	–2.71	3.41	–2.08	–1.63
Ni@b_66_	1.04	–3.13	4.72	–7.62	0.20	–4.37	47.6	–1.38	–0.99

For isolated B_12_N_12_, the relatively high chemical hardness (η = 2.64 eV) and moderate electrophilicity (ω = 3.15 eV) indicate an electronically stable and weakly reactive system, consistent with its wide band gap (5.29 eV). The cohesive energy (−7.68 eV) confirms its high structural stability, in agreement with previous theoretical reports on BN nanocages.^56,57^ After adsorption, the small variation in η (2.60 eV) and Δ*G*_ads_ of −0.85 eV indicates a spontaneous but moderate interaction. For isolated Ni@b_64_, the very low hardness (η = 0.60 eV) reflects high polarizability and enhanced reactivity, while the high electrophilicity (ω = 12.18 eV) suggests strong electron-accepting capability. Although the cohesive energy (*E*_coh_ = −7.67 eV) remains comparable to that of pristine B_12_N_12_, metal decoration substantially increases reactivity. Upon adsorption, the increase in η (1.07 eV) indicates electronic stabilization of the complex, whereas Δ*H*_ads_ (−2.08 eV) and Δ*G*_ads_ (−1.63 eV) confirm strongly spontaneous chemisorption and greater thermodynamic stability of the adsorbed system.

For isolated Ni@b_66_, the intermediate hardness (η = 1.04 eV) and ω = 4.72 eV indicate moderate reactivity. After adsorption, the marked decrease in η (0.20 eV) and the sharp increase in ω (47.6 eV) are consistent with pronounced electronic reorganization. However, the less negative Δ*G*_ads_ (−0.99 eV) compared to Ni@b_64_ suggests energetically favorable yet comparatively weaker adsorption, more suitable for reversible sensing processes. The high electrophilicity observed after NH_3_ adsorption arises from the pronounced reduction in the chemical hardness of the system, resulting in a more polarized and electronically deformable state. Since the electrophilicity index (ω) is inversely proportional to the chemical hardness (η), small decreases in η lead to a substantial increase in ω. This behavior does not indicate numerical instability but rather reflects an enhanced ability of the adsorption complex to accept electron density and undergo electronic reorganization upon NH_3_ adsorption. The consistency of this result with the DOS/PDOS, CDD, ELF, RDG, and MD analyses further confirms that the high electrophilicity originates from significant adsorption-induced electronic redistribution rather than computational artifacts.

To evaluate the thermodynamic stability of the systems studied, we employed the optimized DFT geometries as the initial configurations for all molecular dynamics simulations. The MD calculations were not intended as an independent structural optimization but rather as a means of evaluating the dynamic stability of the adsorption complexes under finite-temperature conditions. This approach enables a direct comparison between the equilibrium structures predicted by DFT and their structural evolution during the simulations, allowing for the persistence of NH_3_ adsorption to be assessed in the presence of thermal fluctuations. Consequently, the MD results complement the static DFT calculations by verifying whether the optimized adsorption configurations remain stable under conditions that are more closely representative of practical sensing environments. This strategy of complementary application of DFT and MD has been widely used in the literature.^41,58–62^

MD was performed at the GFN-2 xTB level in the NVT ensemble at 298.15 K for 1000 ps, using a 2.0 fs integration time step with dump = 100 and without reassignment of initial velocities. Temperature control was maintained under constant NVT conditions with hydrogen mass repartitioning applied (hmass = 4) to enhance numerical stability. The SHAKE algorithm (shake = 2) was employed to constrain bonds involving hydrogen atoms, and an SCC convergence criterion of 2.0 ensured electronic stability throughout the trajectory.

The stability of the isolated nanocages at high (Figure S3 in the Supporting Information) end room temperature has been investigated end attested by MD in previous work.^41^ The energy–time profiles shown in Figure 9 display the total energy evolution for B_12_N_12_–NH_3_, Ni@b_64_–NH_3_, and Ni@b_66_–NH_3_ systems. All systems exhibit controlled thermal fluctuations around stable mean energy values, with no significant energy drift, confirming thermodynamic equilibrium. The systems reach stable fluctuations following the initial relaxation stage—showing no continuous energy drift and a maximum total energy variation of 0.03 eV—which indicates thermodynamic equilibrium. The constant energy fluctuation observed in the plots arises from the movement of the NH_3_ molecule adsorbed at the metal site; however, the adsorption remains stable from the outset and throughout the entire trajectory. The Ni@b_64_–NH_3_ system shows the most stable average energy and comparatively smaller relative fluctuations, indicating stronger and more persistent interaction between NH_3_ and the metallic center. Ni@b_66_–NH_3_ also maintains structural integrity, though with slightly larger fluctuations, consistent with weaker adsorption. In all cases, ammonia remains adsorbed throughout the simulation, highlighting the dynamic stability of adsorption under ambient conditions.

**Figure 9:**
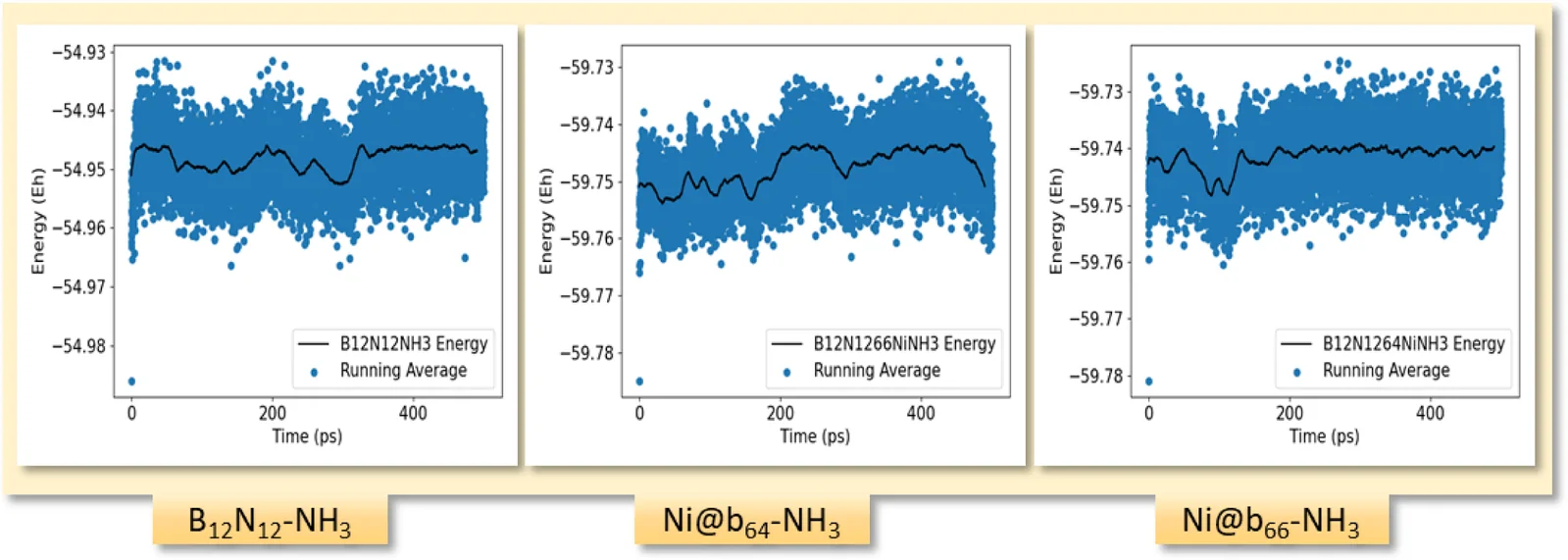
MD analysis of the B_12_N_12_–NH_3_, Ni@b_64_–NH_3_ and Ni@b_64_–NH_3_ systems (total energy systems in blue and average energy in black).

### Charge and Interaction Analysis

Charge transfer upon NH_3_ adsorption was consistently characterized using CDD (Figure 10), CDA (Table 6), and Mulliken analyses, confirming a donor–acceptor mechanism dominated by electron donation from NH_3_ to the nanocage. The CDD maps show charge depletion on NH_3_ and accumulation at the adsorption site, particularly in the Ni–N region for decorated systems. Quantitatively, CDA reveals net charge transfer values of −0.187 |e| (B_12_N_12_–NH_3_), −0.120 |e| (Ni@b_64_–NH_3_), and −0.082 |e| (Ni@b_66_–NH_3_), with back-donation contributions of 0.192, 0.143, and 0.110, respectively. The donation terms increase slightly from 0.005 (B_12_N_12_) to 0.023 and 0.027 in Ni@b_64_ and Ni@b_66_, indicating enhanced metal–ligand interaction.

**Figure 10:**
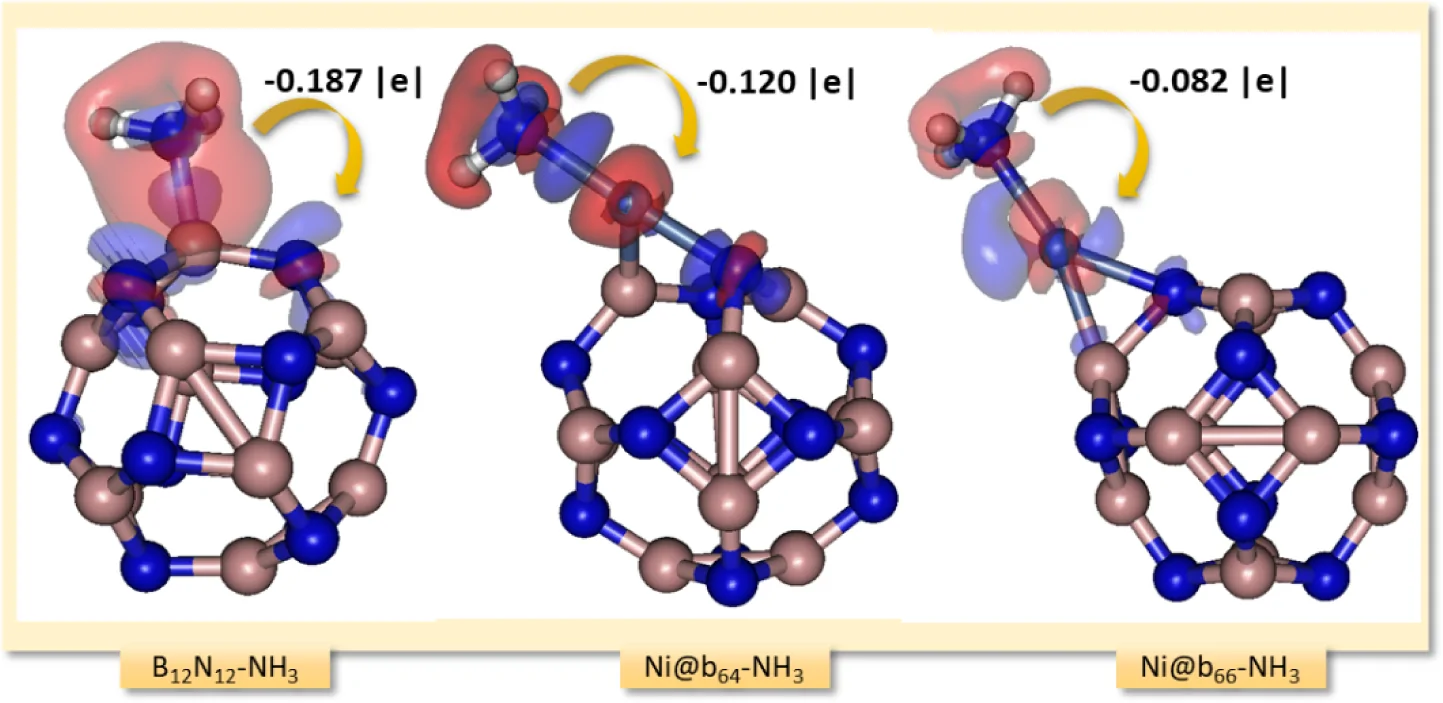
Charge density difference (CDD) calculated for B_12_N_12_–NH_3_, Ni@b_64_–NH_3_ and Ni@b_66_–NH_3_ systems.

**Table 6:** CDA Analysis of the B_12_N_12_–NH_3_, Ni@b_64_–NH_3_ and Ni@b_66_–NH_3_ Systems: Donation, Back-Donation, Transferred Charge, and Repulsive Charge

Systems	Donation	Back-Donation	Transf. Charge	Repulsive Polarization
B_12_N_12_–NH_3_	0.005	0.192	–0.187	–0.434
Ni@b_64_–NH_3_	0.023	0.143	–0.120	–0.229
Ni@b_66_–NH_3_	0.027	0.110	–0.082	–0.086

These trends are supported by Mulliken charges on NH_3_ (+0.320, +0.220, and +0.219 |e|), confirming reduced electron donation upon metal decoration. Ni@b_64_ exhibits stronger charge transfer and polarization (repulsive term = −0.229), consistent with its higher adsorption energy, whereas Ni@b_66_ shows lower polarization (−0.086) and weaker interaction. This progressive reduction in charge transfer and polarization supports a transition from strong chemisorption to more reversible adsorption, reinforcing the suitability of Ni@b_66_ for sensing applications.

ELF analysis provides direct insight into the bonding character at the adsorption interface, as presented in Figure 11. For pristine B_12_N_12_, high ELF values (≈0.80–0.95) localized along B–N bonds and nitrogen lone pairs confirm its polar covalent framework. Upon NH_3_ adsorption, the emergence of intermediate localization (ELF ≈ 0.55–0.65) at the interface indicates a donor–acceptor interaction without the formation of a fully developed covalent bond. In Ni-decorated systems, the Ni–N region exhibits enhanced localization, particularly for Ni@b_64_ (ELF ≈ 0.65–0.75), indicating increased electron pairing and partial covalent character associated with metal–ligand coordination. In contrast, Ni@b_66_ shows weaker localization (≈0.55–0.65), consistent with a more electrostatic interaction. These results indicate that interaction strength and covalent contribution decrease in the order Ni@b_64_–NH_3_ > B_12_N_12_–NH_3_ > Ni@b_66_–NH_3_, supporting the balance between stability and reversibility required for sensing applications.

**Figure 11:**
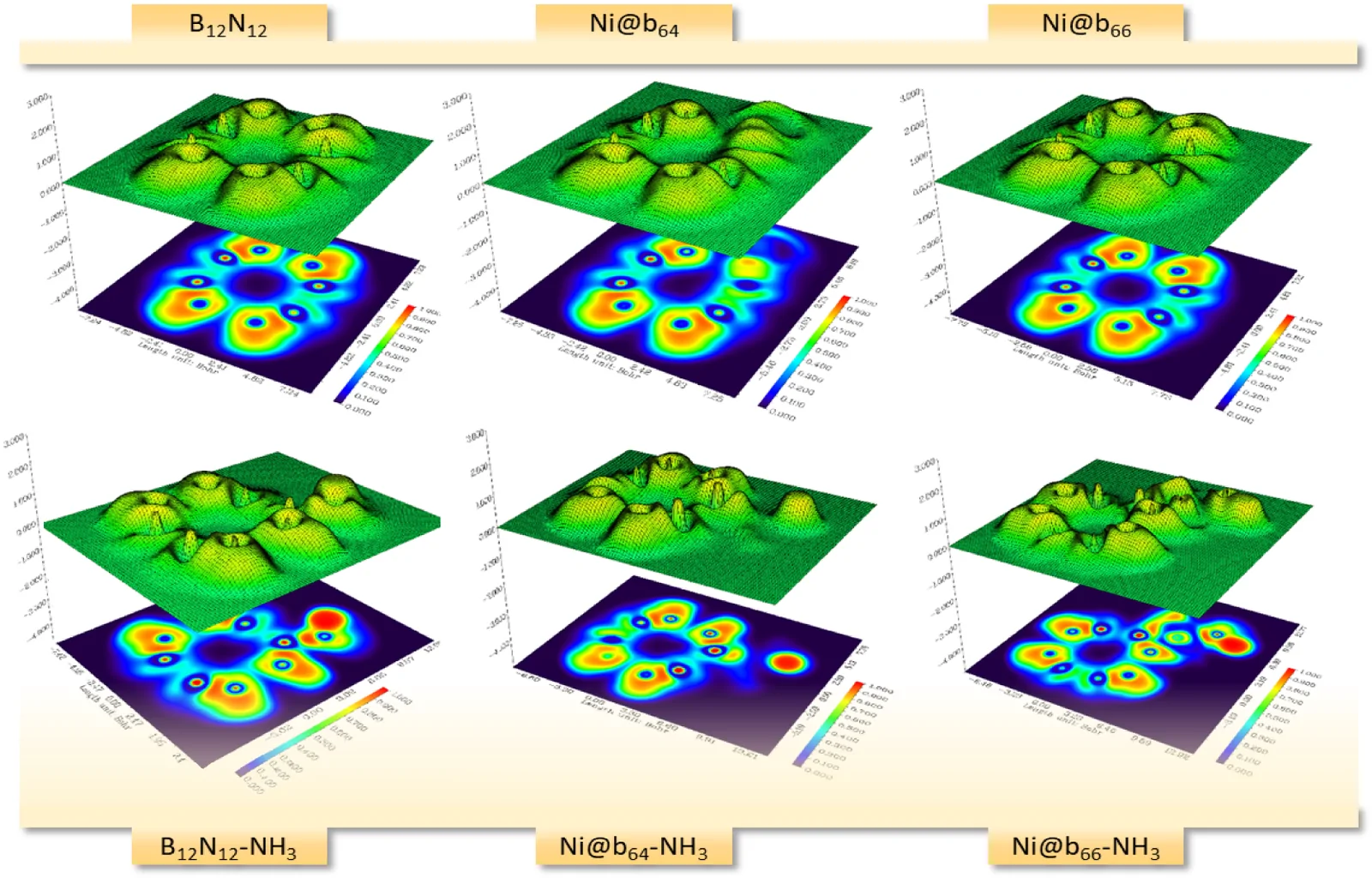
ELF plot analysis for B_12_N_12_, Ni@b_64_ and Ni@b_66_ with NH_3_ systems.

Figure 12 presents the reduced density gradient (RDG) isosurfaces together with the RDG vs. sign­(λ_2_)­ρ scatter plots derived from the NCI analysis, enabling a detailed characterization of the noncovalent interactions governing NH_3_ adsorption on the nanocages. For the B_12_N_12_–NH_3_ system, the RDG isosurfaces are dominated by green regions located at the gas–cage interface, which are characteristic of weak dispersive (van der Waals) interactions. This interpretation is corroborated by the RDG distribution, where the majority of points are concentrated near sign­(λ_2_)­ρ ≈ 0, indicating a predominantly noncovalent interaction regime. In contrast, the Ni-decorated systems exhibit additional blue–green features localized in the Ni–N region, reflecting enhanced attractive interactions arising from coordination between the ammonia lone pair and the Ni center. Consistently, the RDG plots reveal a higher population of points in the negative sign­(λ_2_)­ρ domain for Ni@b_64_–NH_3_, indicating stronger stabilizing interactions, whereas Ni@b_66_–NH_3_ displays an intermediate distribution. These findings align with the ELF analysis, where the absence of a disynaptic basin excludes classical covalent bonding, while increased ELF localization near the Ni–N interface indicates a donor–acceptor interaction with partial covalent character. Collectively, the NCI and ELF analyses establish the interaction strength order as Ni@b_64_–NH_3_ > Ni@b_66_–NH_3_ > B_12_N_12_–NH_3_.

**Figure 12:**
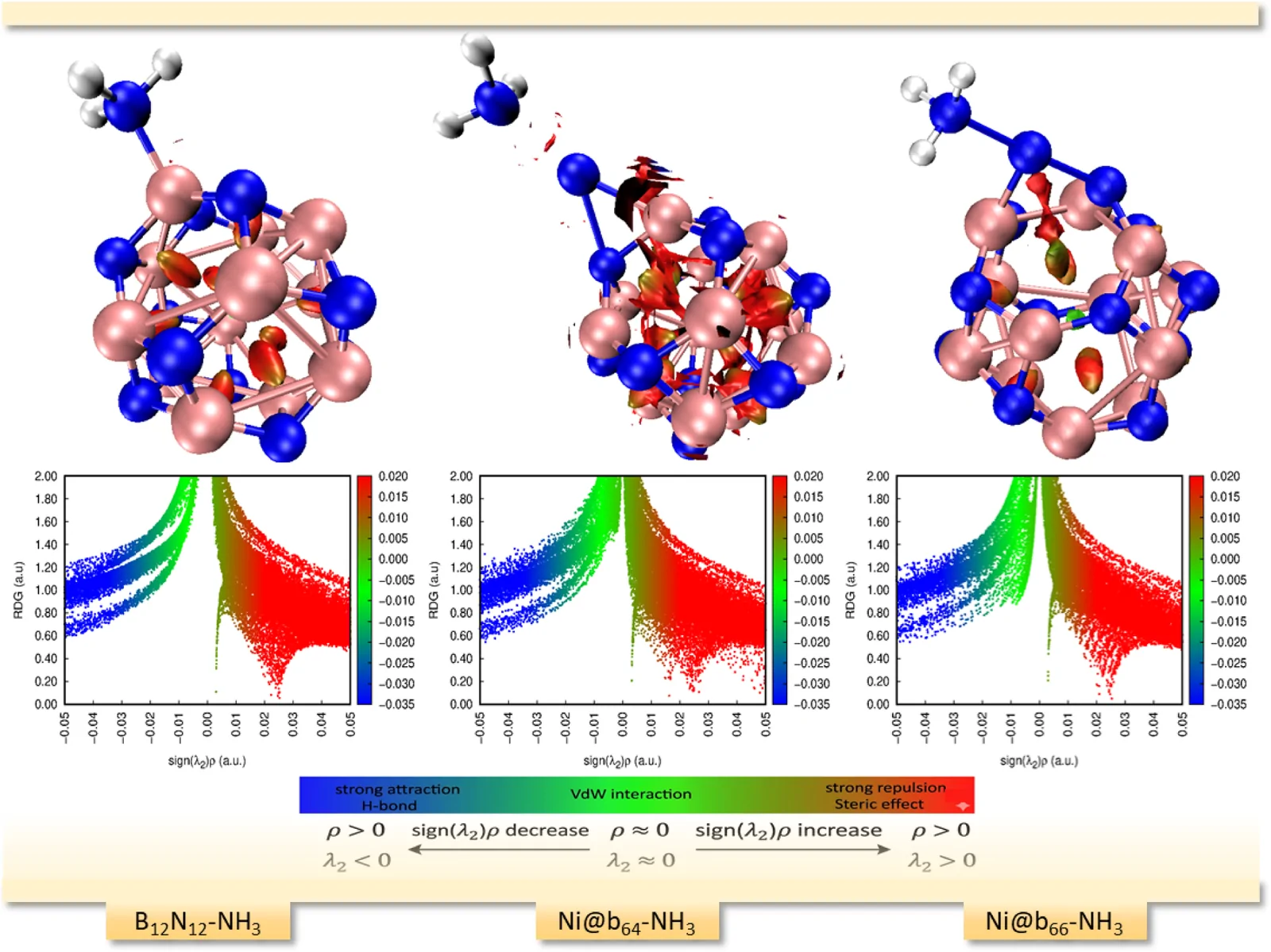
NCI and RDG plot analysis for B_12_N_12_–NH_3_, Ni@b_64_–NH_3_ and Ni@b_66_–NH_3_ systems.

The analysis of the CDA, CDD, Mulliken charges, ELF, and RDG results explains why pristine B_12_N_12_ exhibits greater net charge transfer from NH_3_ than Ni@b_64_ (−0.187 vs. −0.120 |e|). Figure 6 shows that, in isolated B12N12, the frontier orbitals are regularly distributed over the nanocage, which may justify the greater charge reception from NH_3_. On the other hand, although the pristine cage receives more electrons, Ni decoration significantly enhances orbital hybridization, electronic polarization, and the donor–acceptor interaction through the participation of Ni 3d orbitals. Consequently, adsorption strength is governed not only by net charge transfer but also by orbital overlap, partial covalent character, and local electronic redistribution around the Ni center.

The combined structural, electronic, and energetic analyses establish a unified mechanism governing NH_3_ adsorption on Ni-decorated nanocages. The b64 and b66 adsorption sites distinctly modify the Ni 3d electronic states near the Fermi level, resulting in different degrees of orbital hybridization between the Ni 3d orbitals and the nitrogen lone pair in NH_3_, as evidenced by the PDOS and HOMO/LUMO analyses. These electronic differences are directly reflected in the adsorption characteristics. The Ni@b64 system exhibits greater electronic polarization, stronger orbital overlap, and a more pronounced partial covalent character of the Ni–N bond, as confirmed by the CDA, ELF, and RDG analyses, leading to stronger adsorption (*E*_ads_ = −1.69 eV) and, consequently, an extremely long recovery time. In contrast, the b66 site promotes lower charge transfer, reduced electronic polarization, and a more moderate donor–acceptor interaction, resulting in weaker adsorption (*E*_ads_ = −0.82 eV). This intermediate interaction strength preserves significant electronic modulation (Δ*E*_gap_ = 42.85%) while lowering the desorption barrier, yielding a short recovery time of only 3.74 s. These findings demonstrate that the sensing performance is governed by a direct relationship between orbital hybridization, charge redistribution, adsorption strength, and recovery time, with the b66 configuration providing the optimal balance between sensitivity and reversibility.

### Optical and Selectivity Analysis

TD-DFT calculations were employed to perform an optical analysis of the systems and to plot the UV–Vis absorption bands of the nanocages before and after NH_3_ adsorption. NH_3_ adsorption induces pronounced modulation in the optical response of the nanocages, as evidenced by the UV–vis spectra in Figure 13. Pristine B_12_N_12_ exhibits absorption in the deep UV region (166–186 nm), consistent with intrinsic π → π* transitions and its wide band gap (5.29 eV). Ni decoration contributes significantly to the frontier orbitals, as shown in Figure 6, and introduces new transitions at longer wavelengths (255–695 nm), reflecting the contribution of Ni 3d states and the reduction of the band gap (1.19 and 2.08 eV, respectively). Upon NH_3_ adsorption, noticeable spectral shifts occur because it drives further redistribution of electron density and hybridization between the ammonia nitrogen’s lone pair and Ni 3d states, particularly for Ni@b_64_ (255 → 283 nm), 695 nm to 381 nm, indicating a significant increment in energy gap (2.15 eV) and electronic reorganization. In Ni@b_66_, the shifts are more moderate (262 → 274 nm; 557 → 554 nm), consistent with its small gap change (2.08 → 2.03 eV), a small charge transfer (+0.082 |e|), and balanced interaction strength (corroborated by ELF analysis). These bathochromic shifts and changes in intensity confirm that NH_3_ adsorption directly perturbs frontier orbitals, enabling optical detection through measurable wavelength variations. These results demonstrate that the optical response is directly linked to the reorganization of frontier orbitals driven by the NH_3_–Ni interaction, establishing a direct correlation between the adsorption configuration and the observed spectral signature.

**Figure 13:**
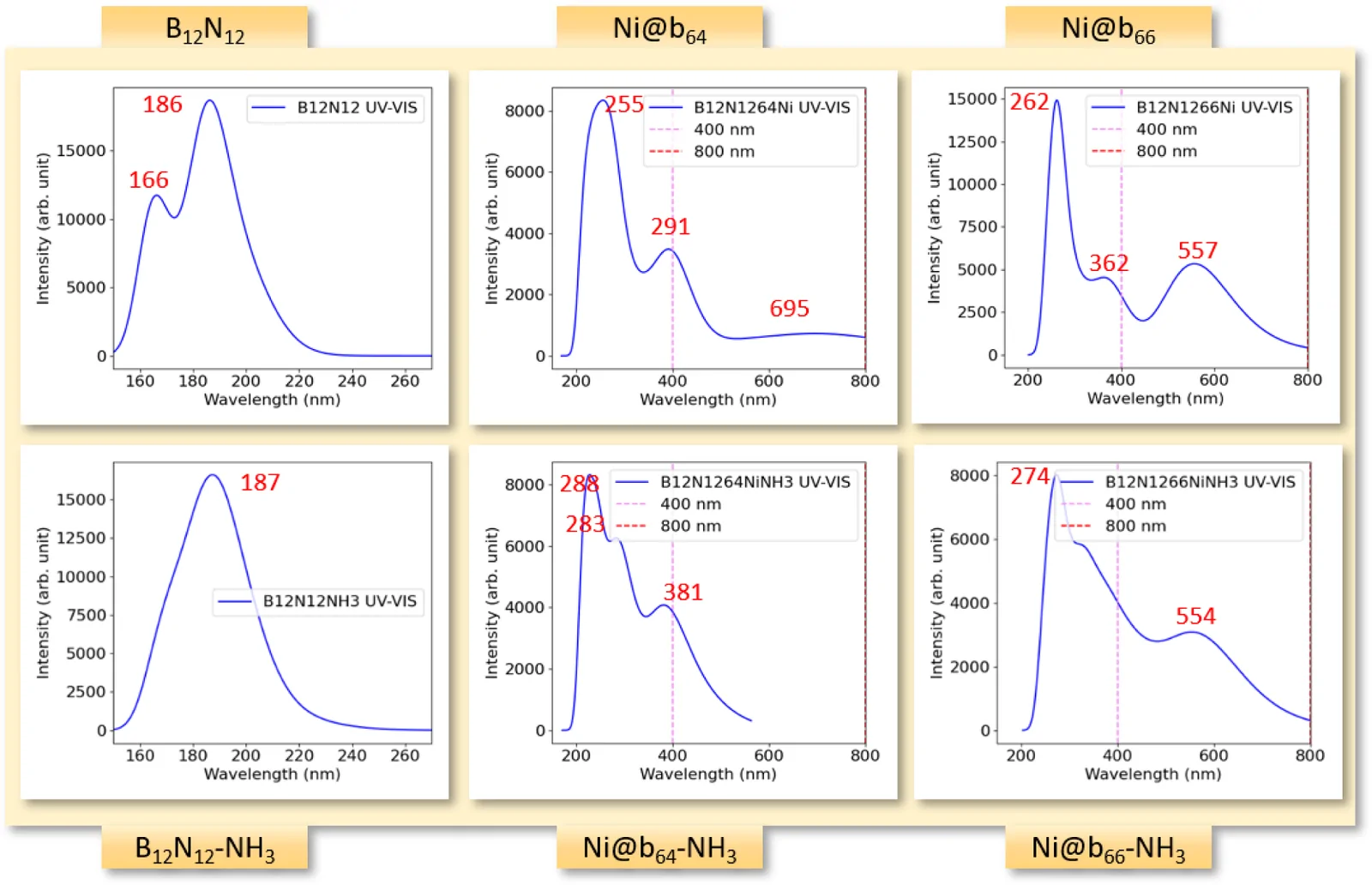
UV–vis spectrum analysis calculated by TD-DFT using B97-3c/6-31G­(d,p).

The data in Table 7 demonstrate that the electronic transitions responsible for the UV–vis spectral peaks are predominantly governed by frontier molecular orbitals. For pristine B_12_N_12_, the absorption bands at 166 nm (*f* = 0.0039) and 186 nm (*f* = 0.140) are mainly characterized by H → L contributions (63% and 32%) and H – 1 → L + 1 transitions (31% and 21%), corresponding to intrinsic π → π* excitations within the BN framework. The introduction of Ni significantly modifies the excitation pattern. In Ni@b_64_, the band at 255 nm (*f* = 0.034) retains dominant H → L character (58%), while the visible transition at 695 nm (*f* = 0.059) is almost purely H → L (90%), indicating direct involvement of Ni 3d-derived frontier states, as observed in the HOMO-LUMO redistribution in the PDOS analysis. For Ni@b_66_, the transitions at 262 and 557 nm exhibit mixed H­(α) → L­(α) and H­(β) → L­(β) contributions, reflecting metal-induced electronic splitting and the open-shell nature of the system, which corroborates a triplet ground state. Upon NH_3_ adsorption, the bands shift to 283 and 381 nm (Ni@b_64_–NH_3_) and to 274 and 554 nm (Ni@b_66_–NH_3_), remaining largely dominated by H → L contributions (>60%). This confirms that NH_3_ adsorption directly modulates the frontier orbitals responsible for the observed optical excitations.

**Table 7:** Electronic Transition Analysis Calculated via TD-DFT Level Using ORCA

System	λ (nm)	*f*	Main Contributions (%)
B_12_N_12_	166	0.0039	H → L (63%), H – 1 → L + 1 (31%)
	186	0.140	H → L (32%), H – 1 → L + 1 (21%)
Ni@b_64_	255	0.034	H → L (58%), H – 1 → L + 1 (21%)
	695	0.059	H → L (90%), H – 1 → L + 1 (7%)
Ni@b_66_	262	0.130	H(α) → L(α) (43%), H(β) → L(β) (14%)
	362	0.056	H(α) → L(α) (81%), H(β) → L(β) (17%)
	557	0.096	H(α) → L(α) (88%), H(β) → L(β) (9%)
B_12_N_12_–NH_3_	187	0.032	H → L (68%), H – 1 → L + 1 (21%)
Ni@b_64_–NH_3_	283	0.022	H → L (61%), H – 1 → L + 1 (26%)
	381	0.041	H → L (70%), H – 1 → L + 1 (20%)
Ni@b_66_–NH_3_	274	0.044	H(β) → L(β) (84%), H(α) → L(α) (3%)
	554	0.052	H(α) → L(α) (93%), H(β) → L(β) (4%)

The calculated electronic parameters and adsorption properties of the Ni@b_66_ nanocage interacting with NH_3_ and interfering gases (H_2_O, CO_2_, O2, CH_4_, N_2_, and H_2_) are summarized in Table 8.^63,64^ NH_3_ adsorption exhibits a moderate adsorption energy (*E*_ads_ = −0.82 eV), accompanied by significant electronic sensitivity (Δgap = 42.8%) and a short recovery time (τ = 3.7 s), which are desirable characteristics for reversible sensing applications. Although H_2_O and CO_2_ ^64^ show larger variations in the electronic gap of the β orbitals (128.6% and 110.7%, respectively), their adsorption energies are less negative (−0.61 and −0.56 eV) than that of NH_3_. This suggests that, despite producing detectable electronic perturbations, the thermodynamic affinity of the nanocage surface for ammonia is stronger, favoring its preferential adsorption and selective detection. In contrast, CH_4_ and H_2_ exhibit extremely weak adsorption energies (0.03 and −0.01 eV) and negligible electronic responses, while N_2_ shows a moderate interaction (*E*_ads_ = −0.35 eV), which is still weaker than that observed for NH_3_. These results indicate that the Ni@b_66_ nanocage exhibits a higher affinity and a more selective electronic response to NH_3_ than common background interfering gases.

**Table 8:** Calculated Values of Energy Gap (*E*_gap_), Electronic Sensitivity (Δgap), Adsorption Energy (*E*_ads_), and Recovery Time (τ) for Ni@b_66_ Nanocage with CH_4_, N_2_, CO_2_, O_2_, H_2_O, and H_2_ Interfering Gases

Systems	Orbital	*E*_gap_ (eV)	Δgap (%)	*S*	*E*_ads_ (eV)	τ
Ni@b_66_–NH_3_	β	0.40	42.8	9.33	–0.82	3.7 s
Ni@b_66_–H_2_0	β	0.64	128.6	3.74	–0.61	1.0 ms
Ni@b_66_–CO_2_	β	0.59	110.7	4.15 × 10^2^	–0.56	151.0 μs
Ni@b_66_–CH_4_	α	2.79	34.1	0.00	0.03	16.2 fs
Ni@b_66_–N_2_	β	0.20	28.6	1.10 × 10^3^	–0.35	42.7 ns
Ni@b_66_–H_2_	α	2.03	2.4	3.74	–0.01	52.0 fs
Ni@b_66_–O_2_	β	1.03	267.9	2.17 × 10^6^	–4.83	>100 h

The electrical conductivity (σ) and the sensing response (*S*) of a semiconductor/sensor follow eq 8:^65^

8 $$\sigma =AT^{3/2}e^{\left(-E_{\mathrm{gap}}/2k_{B}T\right)}$$

9 $$S=\frac{\left|R_{\mathrm{gas}}-R_{\mathrm{pure}}\right|}{R_{\mathrm{pure}}}=\frac{\left|\frac{1}{\sigma_{\mathrm{gas}}}-\frac{1}{\sigma_{\mathrm{pure}}}\right|}{\frac{1}{\sigma_{\mathrm{pure}}}}=\frac{\left|\sigma_{\mathrm{pure}}-\sigma_{\mathrm{gas}}\right|}{\sigma_{\mathrm{gas}}}$$

10 $$S=e^{\left(-\Delta E_{\mathrm{gap}}/2k_{B}T\right)}$$

where *A* (electron/m^3^ K^3/2^) is a constant, *T* is the thermodynamic temperature, *E*_gap_ is the energy gap, and *k*_B_ is the Boltzmann constant. This equation shows that the electrical conductivity of the system will exponentially vary with the *E*_gap_ changes. When estimating the sensing response, the ratio between the conductivities of the adsorbed and pristine systems is considered. In this ratio, the prefactor *A* and the temperature term (*T* ^3/2^) cancel out, leading to eq 10 Therefore, the sensing response depends on the variation of the band gap induced by molecule adsorption. Considering a Ni@b_66_ gap variation of 0.12 eV upon NH_3_ adsorption, the theoretical response at 298.15 K is approximately S ≈ 9.33.

The selectivity of the Ni@b_66_ system was assessed against the interfering gases (H_2_O, O2, CO_2_, CH_4_, N_2_, and H_2_), as summarized in Table 8. H_2_ shows no measurable response, while CH_4_ and N_2_ exhibit only moderate signals (*S* = 3.74). Although H_2_O, O_2_, and CO_2_ induce high responses (*S* = 1.1 × 10^3^, 2.17× 10^6^, and 4.15 × 10^2^, respectively). In contrast, NH_3_ displays an optimal balance between adsorption strength (*E*_ads_ = −0.82 eV), electronic modulation, and directional charge transfer via Ni–N coordination, resulting in a stable and reproducible conductivity response. An isolated analysis of these data suggests that the Ni@b_66_ sensor is inactive for NH_3_ detection in the presence of H_2_O, O_2_, and CO_2_. This combination uniquely enables NH_3_ to exhibit both significant sensitivity and reversibility, confirming Ni@b_66_ as a selective sensor. Thus, sensing performance is not governed solely by Δ*E*_gap_ magnitude but by the interplay of thermodynamic stability, interaction specificity, and dynamic persistence,^66^ as supported by the next analysis performed.

### Competitive Coadsorption Model

Figure 14 presents a competitive coadsorption model. The optimized geometry of the Ni@b_66_ system in the simultaneous presence of NH_3_, H_2_O, and O_2_, simulating a more realistic sensing environment. Initially, all three molecules were positioned 2.40 Å from the Ni active site. After structural relaxation, NH_3_ remained coordinated to the Ni atom through a Ni–N bond of 1.99 Å, with a Mayer bond order of 0.46, while its net Mulliken charge remained nearly unchanged (+0.213 |e|), indicating that the charge-transfer mechanism is preserved. The BSSE-corrected adsorption energy of NH_3_ was −1.00 eV, slightly more favorable than that obtained for isolated adsorption (−0.82 eV). Meanwhile, O_2_ remained strongly bound to the metallic center through a Ni–O bond of 1.78 Å, consistent with its large adsorption energy (−4.83 eV) and extremely long recovery time, suggesting strong passivation of the active site. Nevertheless, even in the simultaneous presence of O_2_ and H_2_O, with the latter interacting more weakly through a Ni–O distance of 2.20 Å, neither species prevented NH_3_ adsorption. These results demonstrate that prior occupation of the active site by water or oxygen does not inhibit NH_3_ recognition, significantly reinforcing the robustness of the proposed sensing mechanism under realistic environmental conditions. This behavior further indicates that the sensing selectivity is not governed solely by active-site occupation, but rather by the ability of the Ni center to accommodate multiple adsorbates while preserving the donor–acceptor interaction with NH_3_, thereby maintaining the electronic response required for detection.

**Figure 14:**
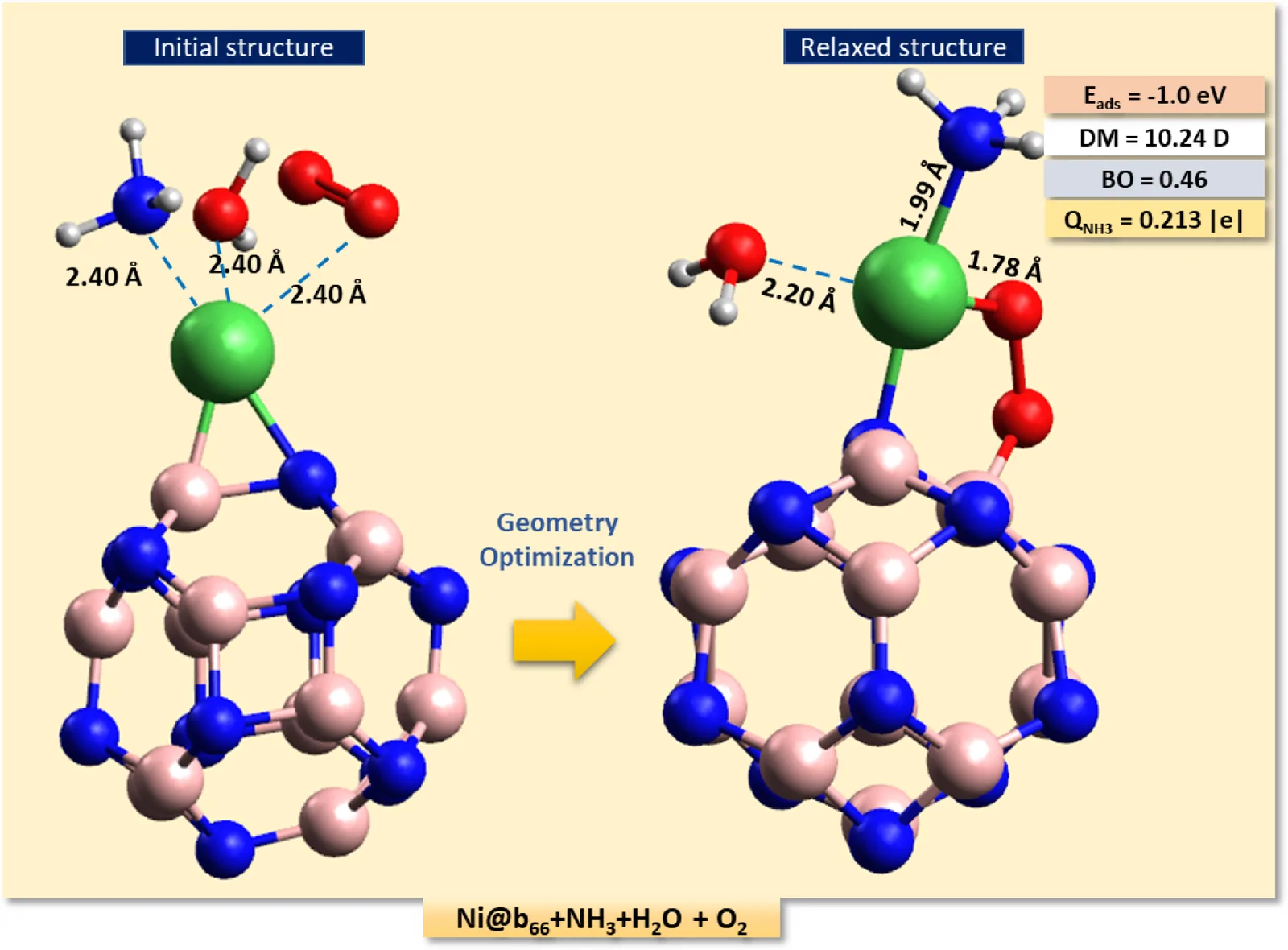
Optimized via DFT of the competitive coadsorption model of O_2_, H_2_O, and NH_3_ molecules on the Ni site of the Ni@b_66_ nanocage.

Subsequently, MD simulations were performed using the xTB/GFN2 method for 300 ps at 298.15 K, considering the simultaneous presence of H_2_, N_2_, CO_2_, CH_4_, NH_3_, H_2_O, and O_2_ around the Ni@b_66_ nanocage. This competitive model enables assessment of the NH_3_ adsorption stability in the presence of multiple interfering species. The initial and final configurations are shown in Figure 15. H_2_ and CH_4_ rapidly diffuse away from the active site, indicating a negligible affinity toward the nanocage, whereas CO_2_ remains in the vicinity of the structure without occupying the Ni active center. In contrast, N_2_, O_2_, H_2_O, and NH_3_ establish stable interactions with the metallic sites throughout the entire simulation. The evolution of the total energy (Figure 16) reveals a gradual stabilization of the system, associated with structural relaxation and the establishment of equilibrium adsorption configurations.

**Figure 15:**
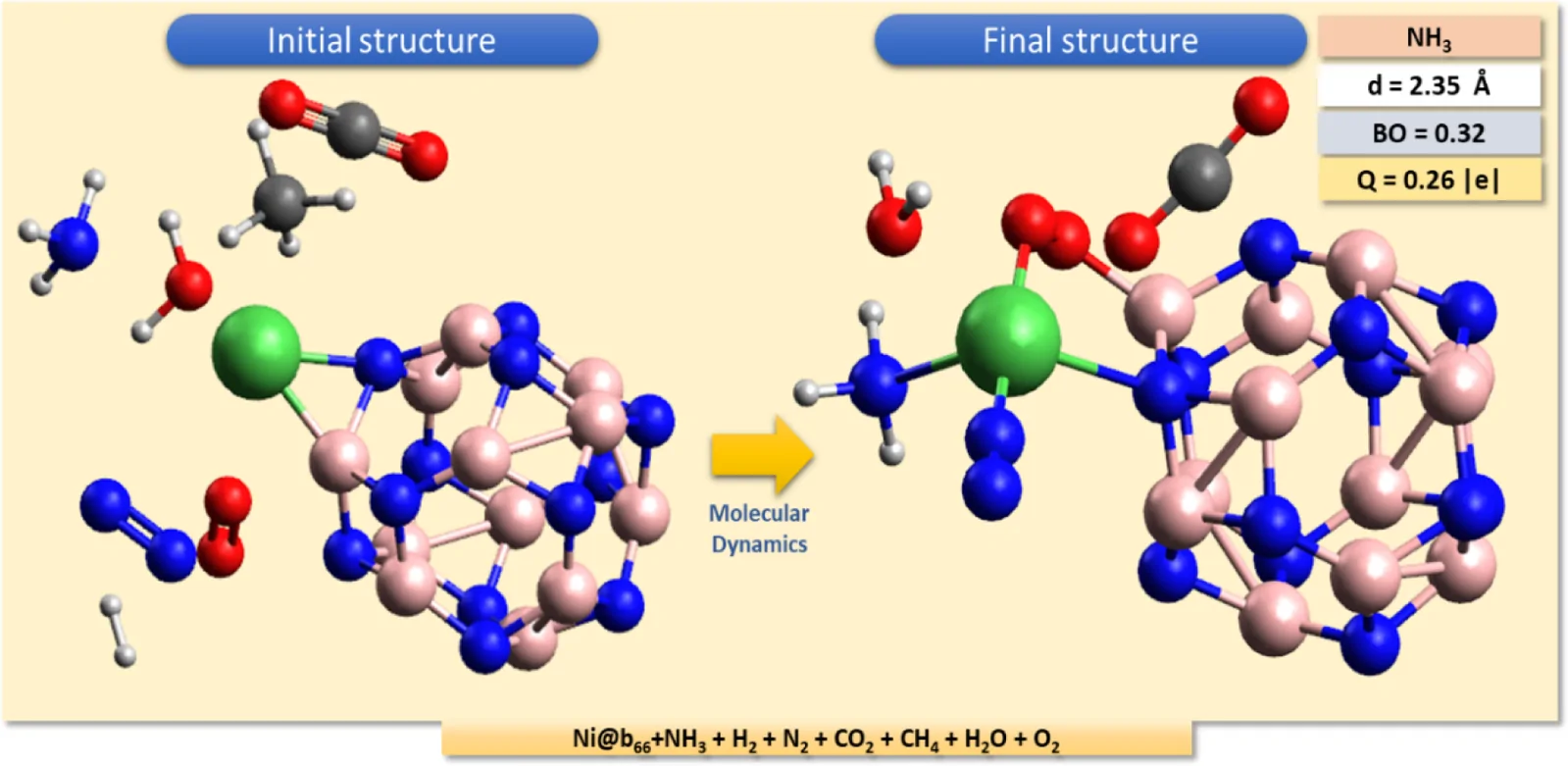
MD simulation of the competitive coadsorption model performed by xTB/GFN-2, with H_2_, N_2_, CO_2_, CH_4_, NH_3_, H_2_O, and O_2_ around the Ni@b_66_ nanocage.

**Figure 16:**
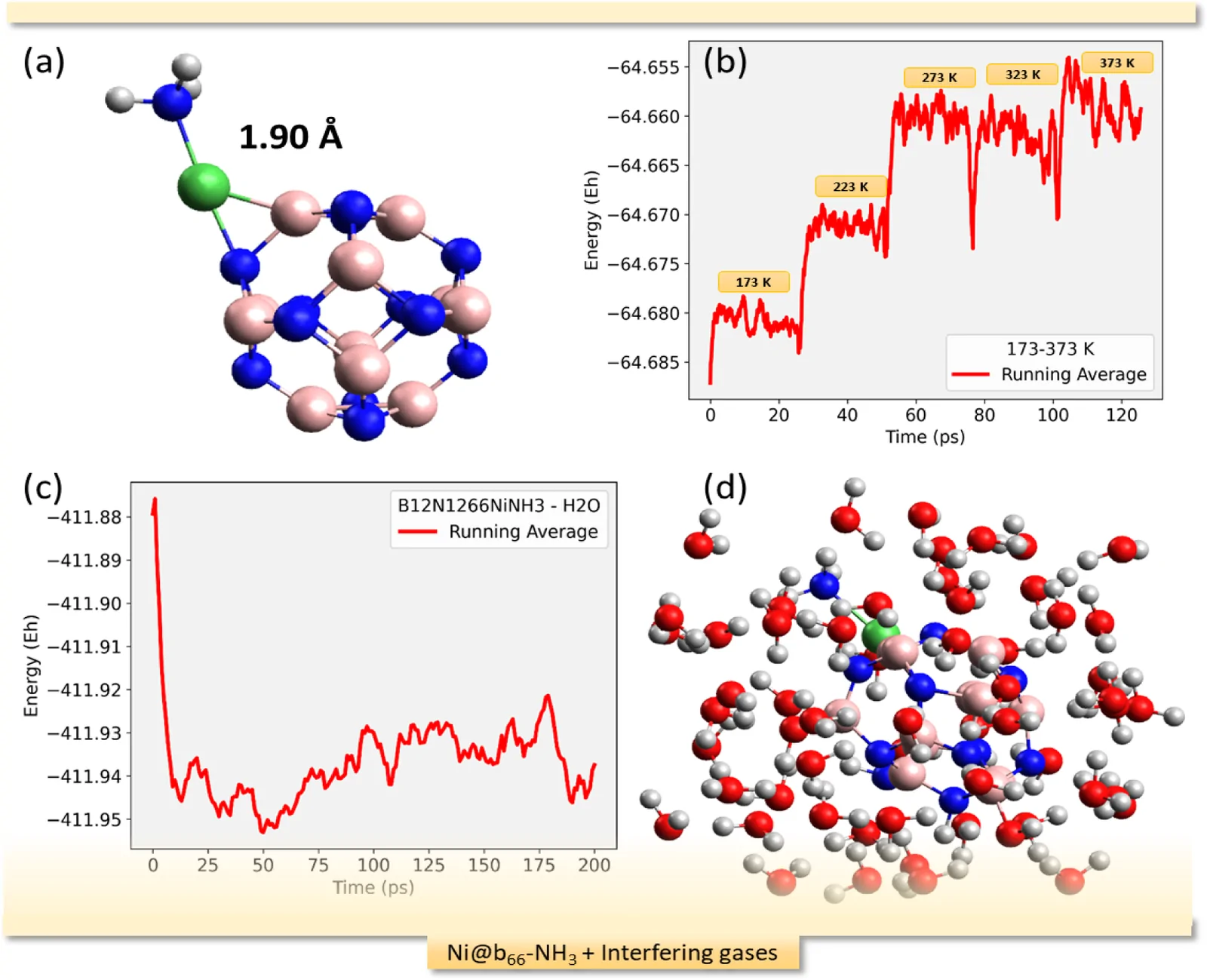
Last Ni@b_66_–NH_3_ geometry (a), energy variation during an MD simulation with a temperature ramp (173–373 K) (b), a system with Ni@b_66_ inserted in a medium with one NH_3_ molecule and 60 water molecules (d), and MD trajectory performed (c).

The structural descriptors obtained at the end of the simulation (Table S2 in the Supporting Information) further support these observations. Although CO_2_ exhibits a significant electronic response when evaluated individually, under competitive conditions it remains only weakly coordinated, with a gas-cage distance of 2.41 Å, a Mayer bond order of 0.59, and a low charge transfer of 0.08 |e|. Likewise, N_2_ approaches the Ni center (1.65 Å) but exhibits only 0.06 |e| of charge transfer, indicating limited electronic interaction. In contrast, O_2_ forms a strong chemisorptive interaction through a bridge configuration involving the Ni atom and a neighboring B atom, characterized by a Ni–O distance of 1.82 Å, a Mayer bond order of 0.88, and a charge transfer of −0.24 |e|, corroborating partial passivation of the active site. Nevertheless, NH_3_ remains coordinated to the Ni atom throughout the simulation, exhibiting a Ni–N distance of 2.35 Å, a Mayer bond order of 0.32, and the largest electron donation to the nanocage (0.26 |e|). These findings demonstrate that even in a competitive, multicomponent environment, the presence of strongly interacting species does not suppress NH_3_ adsorption, confirming the robustness of the proposed sensing mechanism.

### Adsorption Analysis under Varying Temperature and High Humidity

In industrial environments, NH_3_ sensors can be subjected to extreme conditions such as high temperatures or high humidity. The stability of the Ni@b_66_–NH_3_ system was assessed by MD simulations, as shown in Figure 16. Under a temperature ramp (173–373 K), the energy exhibits stepwise variations with stable averages in each regime (Figure 16b), indicating thermodynamic equilibrium. No NH_3_ desorption is observed across the entire range, confirming robust thermal stability of the adsorption complex (Figure 16). Under high-humidity conditions,^40,67,68^ simulations performed in a water box containing 60 H_2_O molecules (Figure 16) show rapid equilibration followed by small energy fluctuations around a stable mean value (≈−411.94 Eh, Figure 16). Throughout the trajectory, NH_3_ remains coordinated to the Ni center, while water molecules only reorganize around the nanocage without disrupting the interaction. These results demonstrate that the Ni@b_66_–NH_3_ interaction is resilient to both thermal variation and water interference.

Representative structures extracted from MD trajectories at 173, 273, and 373 K were further analyzed by single-point DFT calculations to evaluate the stability of the Ni@b_66_–NH_3_ complex (Table S3). Only minor variations were observed in the Ni–N bond length (1.90–1.97 Å), Mayer bond order (0.47–0.50), charge transfer (0.220–0.232 |e|), and HOMO-LUMO gap (0.21–0.24 eV), confirming the stability of both the adsorption configuration and the sensing mechanism. Furthermore, the robustness of the system was validated through competitive coadsorption (NH_3_/H_2_O/O_2_), MD simulations including the major atmospheric and industrial gases and water box. In all cases, NH_3_ adsorption and the donor–acceptor interaction were preserved, demonstrating the robustness of the proposed sensing mechanism under realistic operating conditions.

The sensing performance depends on the balance between adsorption energy (*E*_ads_), interaction distance (*d*), and electronic sensitivity (Δgap), as summarized in Table 9. Materials such as pristine graphene (*E*_ads_ = −0.11 eV; Δgap = 5%)^69^ and carbon nanotubes (−0.27 eV; 15%)^70^ exhibit weak interactions, limiting sensitivity. BN-based and doped oxide systems display moderate improvements, such as Zn_12_O_12_ (−0.78 eV; 41.3%).^71^ The proposed Ni@b_66_ system presents *E*_ads_ = −0.82 eV, Δgap = 42.8%, and a short interaction distance (1.98 Å), indicating strong electronic coupling between the adsorbate and the surface. Compared to other systems, it combines significant band gap modulation with an adsorption energy suitable for reversible sensing, outperforming materials with very low *E*_ads_ (weak response) or excessively high values (slow recovery). Therefore, when compared with literature results, the balance between thermodynamic stability, adsorption intensity, and electronic sensitivity positions Ni@b_66_ as a promising candidate for resistive NH_3_ sensing applications.

**Table 9:** Comparison of Sensing Materials for the Detection of Ammonia (NH_3_)

Material	Functional/Basis Set	*E*_ads_ (eV)	*d* (Å)	Δgap (%)	Ref
Graphene	PBE-GGA	–0.11	3.25	5.0	^69^
CNT	B3LYP/6-31G(d)	–0.27	2.89	15.0	^72^
CNT	PBE	–0.32	2.71	18.0	^73^
B_12_N_12_	B3LYP/6-31G(d)	–0.41	2.37	18.6	^74^
BN nanosheet	PBE	–0.38	2.42	22.0	^75^
Al_12_N_12_	B3LYP/6-31G(d)	–0.59	2.14	27.4	^76^
N-doped Graphene	PBE	–0.46	2.23	28.0	^77^
B_12_P_12_	B3LYP/6-31G(d)	–0.52	2.29	29.7	^78^
Phosphorene	PBE	–0.41	2.36	30.0	^79^
Al_12_N_12_	B3LYP/6-31G(d)	–0.57	2.16	30.2	^80^
B-doped Graphene	PBE	–0.58	2.1	32.0	^81^
Al_12_N_12_	PBE/DNP	–0.62	2.19	33.8	^20^
WS_2_ monolayer	PBE	–0.49	2.51	34.0	^82^
Al_12_P_12_	PBE/DNP	–0.67	2.18	35.4	^20^
Sn_12_O_12_	PBE/DNP	–0.65	2.28	36.5	^20^
MoS_2_ monolayer	PBE-D2	–0.52	2.45	38.0	^83^
Al_12_Si_12_	ωB97XD/6-31G(d)	–0.69	2.21	38.9	^84^
g-C_3_N_4_ nanosheet	B3LYP	–0.61	2.17	41.0	^71^
Zn_12_O_12_	B3LYP/6-31G(d)	–0.78	2.12	41.3	^85^
Ni@b_66_	B97-3c/6-31G(d.p)	–0.82	1.98	42.8	This work

Compared with recent studies on nanomaterial-based gas sensors, the present work extends the sensor performance evaluation by combining conventional electronic analyses with validation under more realistic conditions, including molecular dynamics simulations in explicit water, competitive NH_3_/H_2_O/O_2_ adsorption, and multicomponent gas-atmosphere simulations. This integrated approach provides a more comprehensive assessment of the stability, selectivity, and robustness of the sensing mechanism, thereby increasing the reliability of theoretical predictions for practical sensing applications. From an application perspective, the Ni@b_66_ nanocage can be envisioned as a potential chemiresistive sensing material, where NH_3_ adsorption induces electron donation to the Ni center, modulating the band gap and the electrical conductivity. A practical implementation involves a thin-film layer deposited on an insulating substrate with interdigitated electrodes, enabling the resistance-based detection of NH_3_ through adsorption-induced conductivity changes.

The proposed scheme, shown in Figure 17, illustrates the possible translation of the Ni@b_66_ system from the theoretical level to a functional chemiresistive sensor architecture. The nanocage is deposited as an active film on an insulating substrate, connected to interdigitated gold electrodes, ensuring efficient conduction and chemical stability (Figure 17a). The selective interaction between NH_3_ and the Ni site (Figure 17b), evidenced by Ni–N coordination (∼1.90 Å) and charge transfer (−0.082 |e|), induces significant modulation of the electronic structure, reflected in the band gap variation (Δ*E*_gap_ = 42.8%) (Figure 17c). This perturbation is directly translated into a measurable change in the film’s electrical conductivity (Figure 17d). The presence of reversible adsorption (τ = 3.74 s) further supports its suitability for real-time applications. Additionally, the highlighted selectivity toward interfering gases shows that the response is governed by specific and directional interactions. Overall, the scheme effectively suggests a mechanism, device architecture, and sensing response, reinforcing the material’s practical applicability.

**Figure 17:**
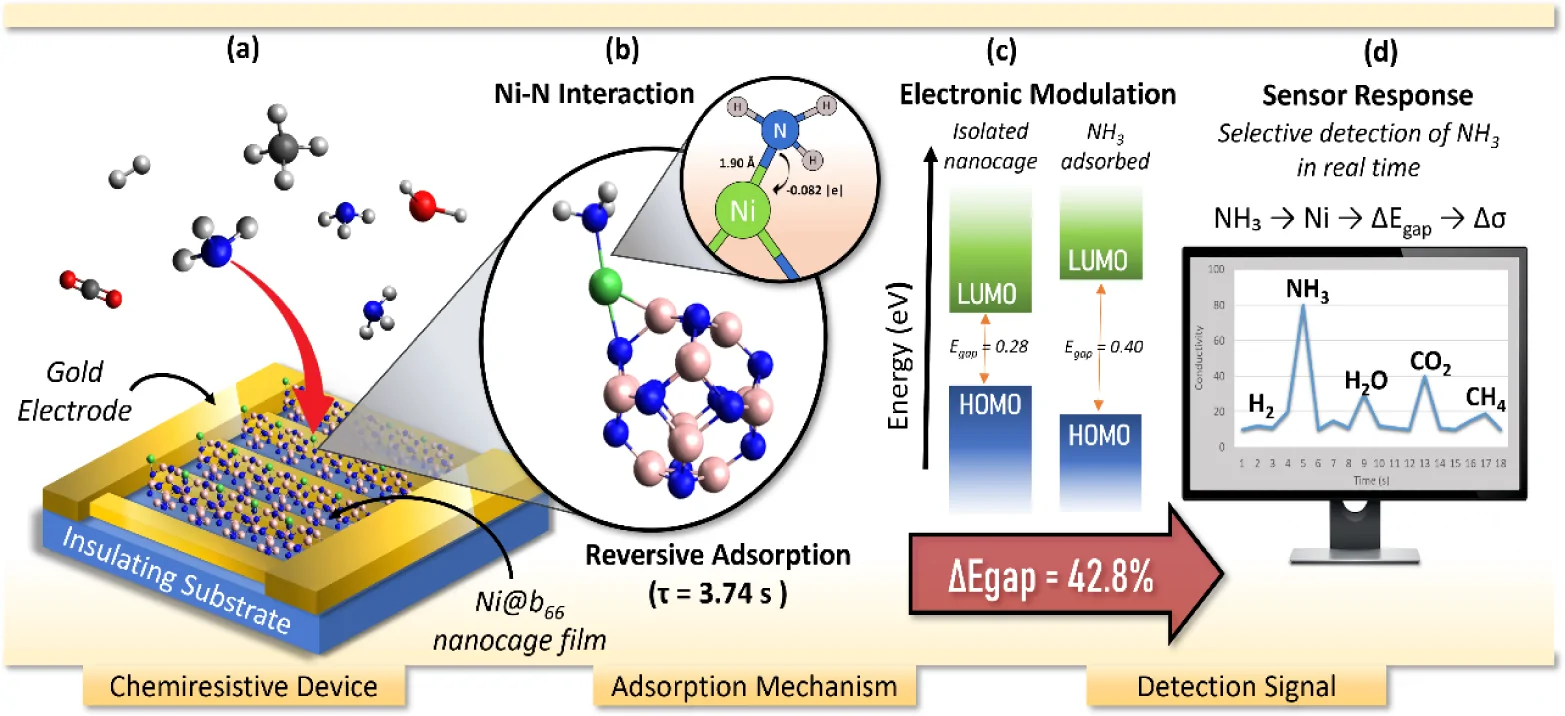
Comprehensive representation of the chemiresistive sensing mechanism, linking the Ni–N interaction to conductivity modulation. Representation of the chemiresistive device (a), NH_3_ adsorption (b), electronic modulation (c), and signal (d).

## Conclusion

This theoretical study established a comprehensive understanding of NH_3_ sensing on Ni-decorated B_12_N_12_ nanocages (Ni@b_64_, Ni@b_66_, Ni@(B), Ni@(N), Ni@(r4), and Ni@(r6)), identifying the Ni@b_66_ configuration, through DFT calculations, as the most promising for reversible ammonia detection. While Ni@b_64_ exhibits high affinity toward NH_3_ (*E*_ads_ = −1.69 eV), indicating potential for NH_3_ capture applications, the Ni@b_66_ system combines moderate interaction strength (*E*_ads_ = −0.82 eV), significant band gap modulation (Δgap = 42.8%), and rapid recovery (τ = 3.74 s at 298 K), making it particularly suitable for chemiresistive sensing. PDOS, ELF, CDD, and CDA analyses consistently revealed a donor–acceptor charge transfer mechanism, in which the NH_3_ molecule donates electron density to the Ni center, establishing a partially covalent interaction capable of producing measurable electronic and optical responses without compromising adsorption reversibility. The robustness of this sensing mechanism was further confirmed through molecular dynamics simulations, analyses at different temperatures (173–373 K), competitive adsorption (NH_3_/H_2_O/O_2_), multicomponent gas atmosphere simulations (H_2_, N_2_, CO_2_, and CH_4_), the negligible influence of atmospheric O_2_ on the NH_3_ sensing response, and explicit water simulation (60 H_2_O molecules), demonstrating high structural stability and strong resistance to environmental interference. Furthermore, benchmark calculations and the quantitative analysis of deviations among different functionals and basis sets confirmed the reliability of the adopted computational methodology. Overall, these findings demonstrate that the Ni decoration site (b_66_) plays a decisive role in sensor performance and provide a consistent mechanistic framework for the rational design of boron nitride-based nanomaterials for selective and reliable NH_3_ sensing under realistic operating conditions.

## Supplementary Material



## Notes

Input data include BSSE calculations; benchmark analyses of functionals and basis sets; energies and errors; optimized adsorption structures; dynamic and thermal stability; and complete XYZ coordinates of the studied systems (PDF)
