# Supplementary material for: Ni-Decorated B12N12 Nanocages as High-Performance Platforms for Reversible NH3 Sensing: A Comprehensive DFT Study
Source: Langmuir. 2026 Sep 7;42(39):28784–803. doi: 10.1021/acs.langmuir.6c02176 (PMC13648274; doi:10.1021/acs.langmuir.6c02176)
Supplement: Supplementary file 1 [file la6c02176_si_001.pdf]

Ni-Decorated $B_{12}N_{12}$ Nanocages as High-Performance Platforms for Reversible NH_3 Sensing: A Comprehensive DFT Study

Natanael de Sousa Sousa[1]*

Affiliation

[1] *Universidade Federal do Maranhão, 65080-805, São Luís, MA, Brazil.*

* Corresponding Author. E-mail address: 83.natan@gmail.com

SUPPORTING INFORMATION

Table of Contents

	BSSE equation
Figure S1	Basis set and functional benchmark analysis for $B_{12}N_{12}$-NH_3, Ni@b_{64}-NH_3, and Ni@b_{66}-NH_3 systems.
Table S1	Calculated energies and relative errors for different functionals and basis sets used in the benchmark analysis
Figure S2	Optimized adsorption structures of $B_{12}N_{12}$-NH_3, Ni@b_{64}-NH_3, and Ni@b_{66}-NH_3
Table S2	Cage-gas distances, bond orders, transferred charges, and total energy from molecular dynamics simulations of the competitive co-adsorption model
Table S3	Structural, electronic, and charge-transfer parameters of the Ni@b_{66}-NH_3 system at 173, 273, and 373 K
Table S4	Cartesian coordinates of the optimized nanocage-NH_3 adsorption structures

BSSE equation

The BSSE energy can be calculated by the original Boys and Bernardi formula for the interaction energy between fragments A and B is (equation S1):

$$\Delta E = E_{AB}^{AB}(AB) - E_{A}^{A}(A) - E_{B}^{B}(B) - E_{A}^{AB}(AB) - E_{A}^{AB}(A) + E_{B}^{AB}(AB) - E_{B}^{AB}(B)$$

Here $E_X^Y(Z)$ is the energy of fragment X calculated at the optimized geometry of fragment Y with the basis set of fragment Z. Thus, you need to do a total the following series of calculations:

1. optimize the geometry of the dimer and the monomers with some basis set Z. This gives you $E_{AB}^{AB}(AB)$, $E_A^{AB}(A)$ and $E_B^{AB}(B)$.
2. delete fragment A (B) from the optimized structure of the dimer and re-run the single point calculation with basis set Z. This gives you $E_B^{AB}(B)$ and $E_A^{AB}(A)$.
3. Now, the final calculation consists of calculating the energies of A and B at the dimer geometry but with the dimer basis set. This gives you $E_A^{AB}(AB)$ and E_B^{AB} (AB).

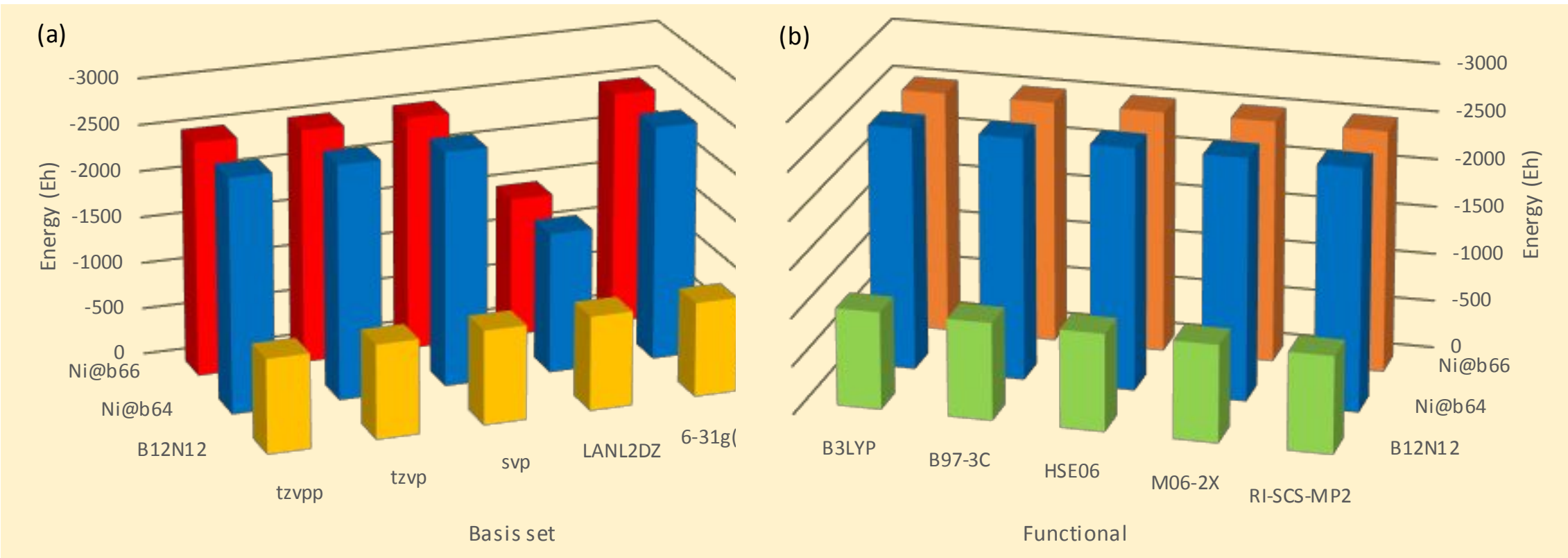

Figure S1 – Different basis set (a) and functional (b) analysis for the $B_{12}N_{12}$-NH_3, Ni@b_{64}-NH_3 and Ni@b_{66}-NH_3 systems.

Table S1 - The calculated values of energy and relative error for different functionals and basis sets employed in the benchmark analysis.

System	Funcional	Basis Set	Energy (Eh)	Error (%)
$B_{12}N_{12}$-NH_3	B97-3C	6-31g(d,p)	-1011.88	
	B97-3C	svp	-1011.18	0.0688
	B97-3C	tzvp	-1012.22	0.0340
	B97-3C	tzvpp	-1012.22	0.0342
	B3LYP	6-31g(d,p)	-1012.23	0.0355
	HSE06	6-31g(d,p)	-1012.45	0.0567
	M06-2X	6-31g(d,p)	-1012.45	0.0567
	RI-SCS-MP2	6-31g(d,p)	-1010.59	0.1269
Ni@b_{64}-NH_3	B97-3C	6-31g(d,p)	-2520.43	
	B97-3C	svp	-2519.75	0.0269
	B97-3C	tzvp	-2521.01	0.0233
	B97-3C	tzvpp	-2521.02	0.0235
	HSE06	6-31g(d,p)	-2519.49	0.0369
	B3LYP	6-31g(d,p)	-2520.27	0.0063

	M06-2X	6-31g(d,p)	-2520.44	0.0006
	RI-SCS-MP2	6-31g(d,p)	-2517.97	0.0976
Ni@b_{66}-NH_3	B97-3C	6-31g(d,p)	-2520.33	
	B97-3C	svp	-2519.66	0.0266
	B97-3C	tzvp	-2520.93	0.0237
	B97-3C	tzvpp	-2520.93	0.0239
	B3LYP	6-31g(d,p)	-2520.2	0.0051
	HSE06	6-31g(d,p)	-2519.44	0.0354
	M06-2X	6-31g(d,p)	-2520.42	0.0033
	RI-SCS-MP2	6-31g(d,p)	-2517.93	0.0952

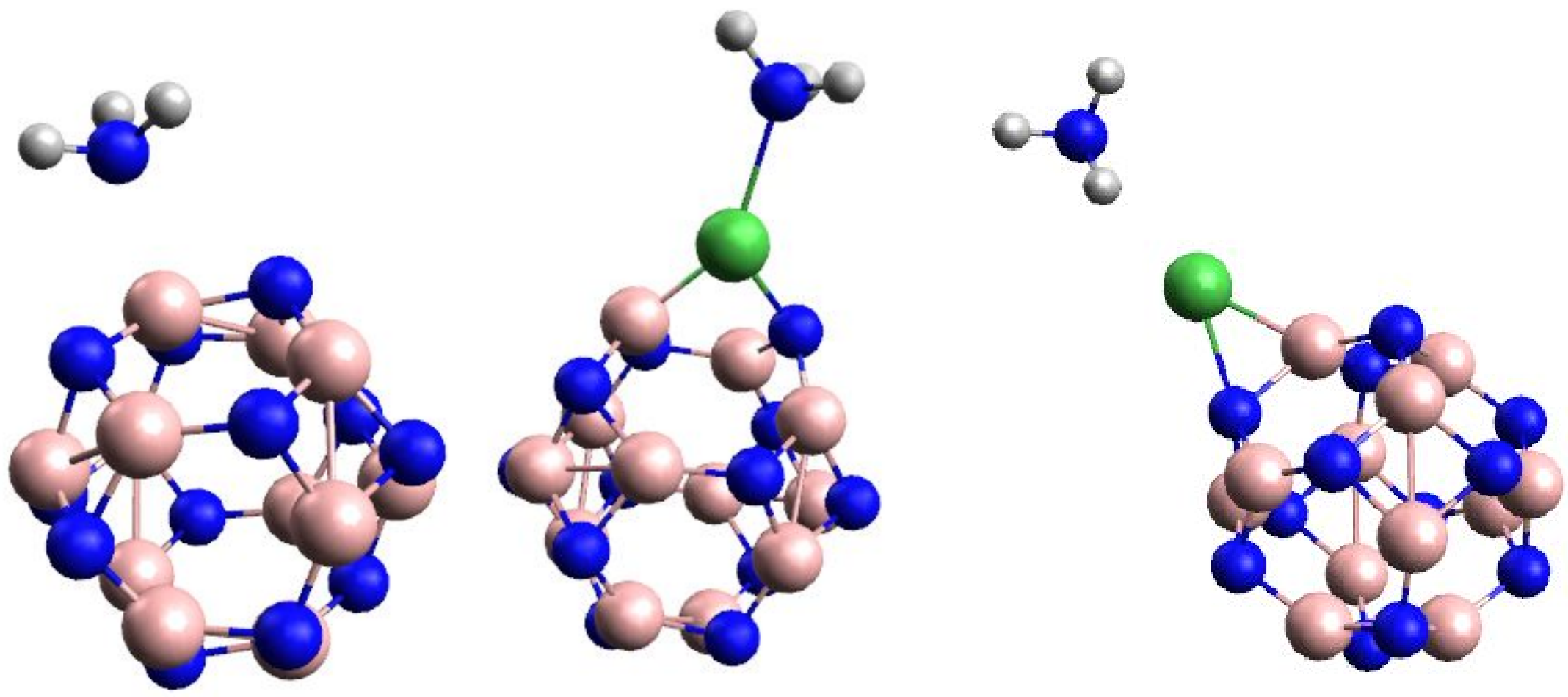

$B_{12}N_{12}$-NH_3 **Ni@b_{64}-NH_3** **Ni@b_{66}-NH_3**

Figure S2 – $B_{12}N_{12}$-NH_3, Ni@b_{64}-NH_3 and Ni@b_{66}-NH_3 adsorption structures

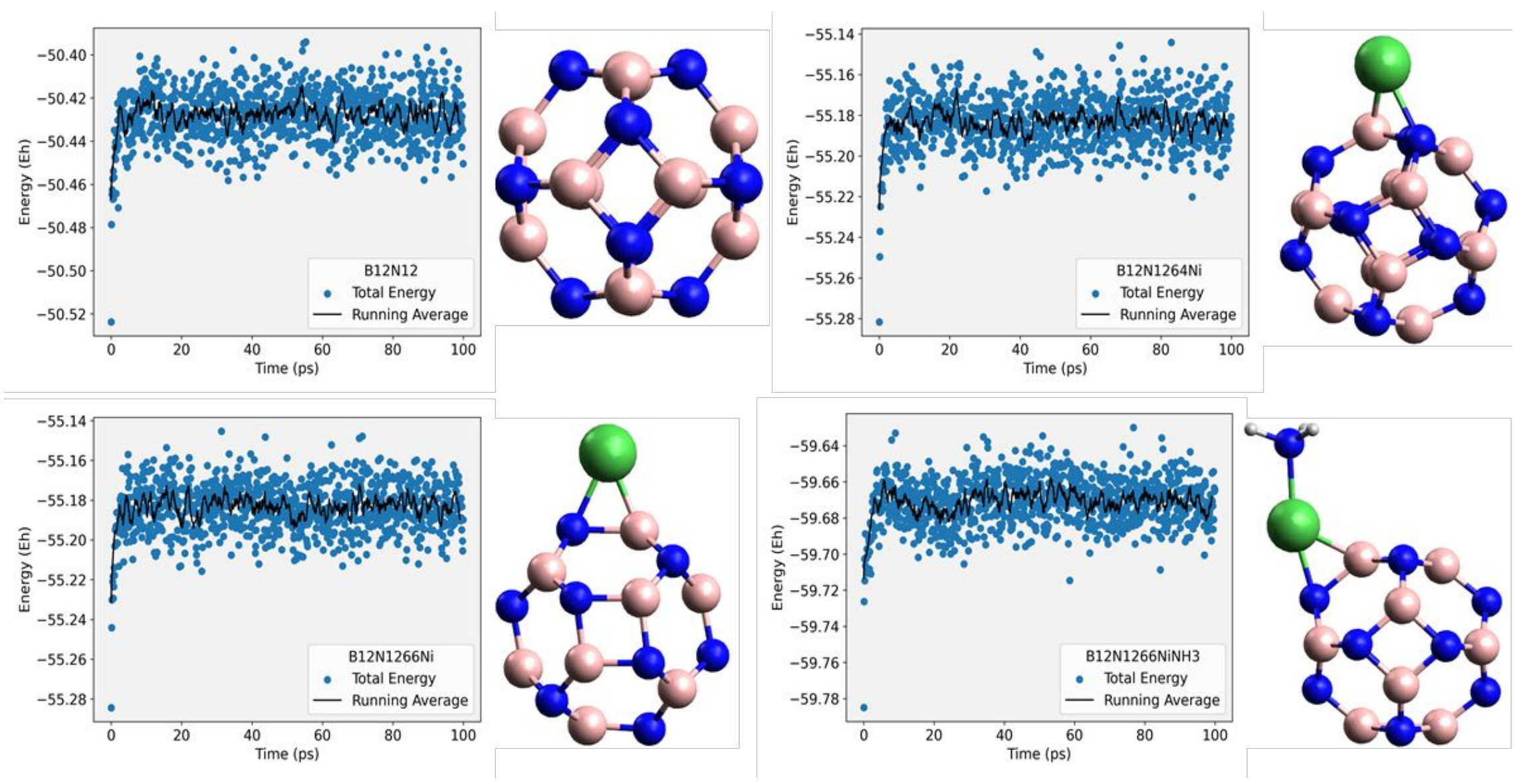

Figure S3 – Stability analysis of the $B_{12}N_{12}$, Ni@b_{64}, Ni@b_{66} and Ni@b_{66}-NH_3 structures at 800 K investigated via molecular dynamics simulations (100 ps - GFN2).

Systems	$d_{gas\text{-}cage}$	B.O.	*Q* |e|
NH_3	2.35	0.32	0.26
H_20	2.17	0.34	0.21
CO_2	2.41	0.59	0.08
CH_4	-	-	0.0
N_2	1.65	0.79	0.06
H_2	-	-	0.0
O_2	1.82	0.88	-0.24

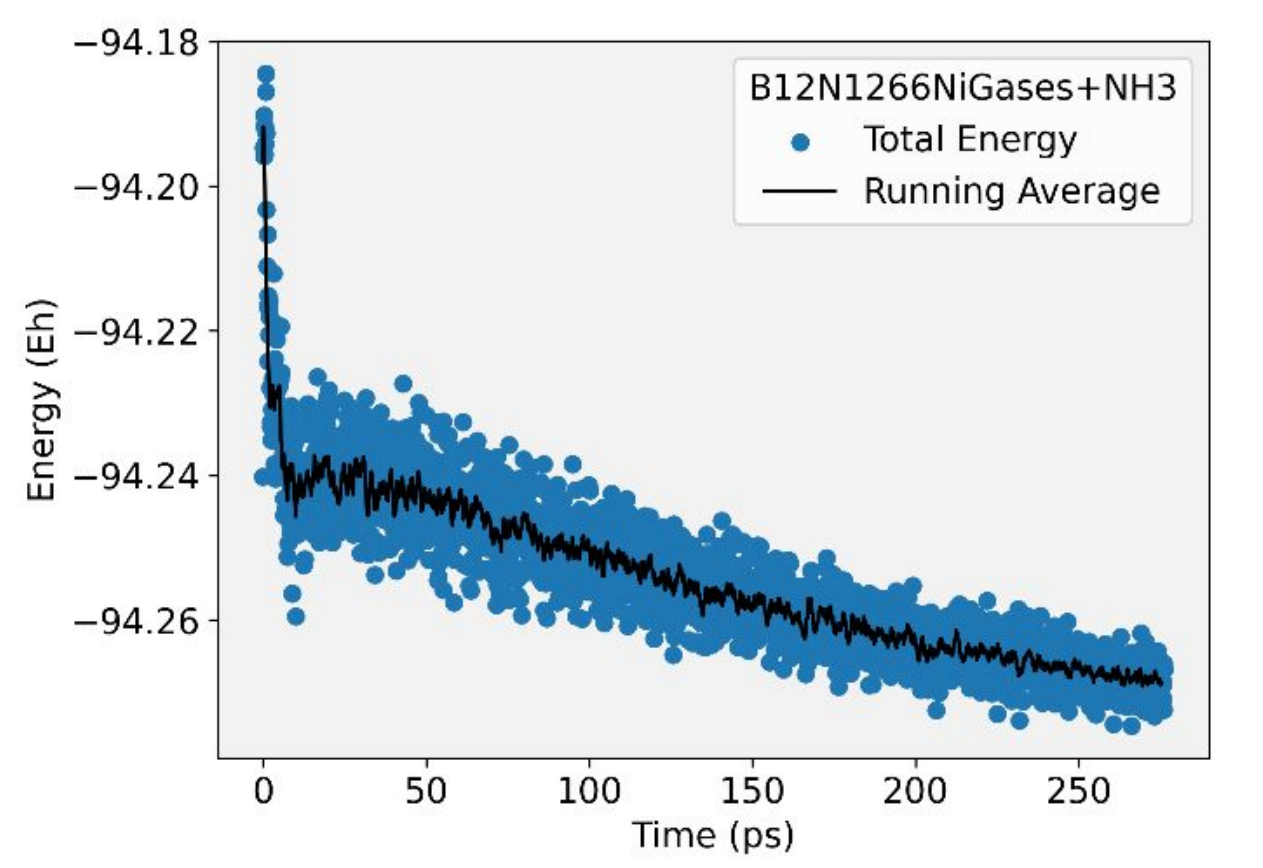

Table S2 – Cage-gas distance ($d_{cage\text{-}gas}$) in Å, bond order cage-gas (B.O.), transferred charge (Q), total energy of MD simulation of the competitive co-adsorption model.

Table S3 – Parameters calculated at 173, 273, and 373K for adsorption Ni@b_{66}-NH_3 system.

**Temperature**	**$d_{Ni\text{-}N}$ (Å)**	**B.O.**	**Q_{NH3} |e|**	**DM (Debye)**	**HOMO-LUMO gap (eV)**
173 K	1.965	0.4704	0.220	8.93653	0.24
273 K	1.900	0.4959	0.232	9.46851	0.21
373 K	1.915	0.4829	0.230	9.65634	0.22

Table S4 - xyz structure of nanocages for NH_3 adsorption.

$B_{12}N_{12}$-NH_3

B	0.94388	0.03451	-1.96757
N	2.11577	0.03111	-1.13478
N	-0.01858	1.15287	-2.11469
N	-0.01103	-1.08746	-2.13431
B	-0.00065	-1.93844	-0.97563
B	-0.01374	1.98300	-0.94093
B	1.96307	-0.93693	-0.02212
B	1.95672	0.97828	-0.00495
N	1.11218	2.14160	0.01074
N	1.12651	-2.10611	-0.02680
B	-0.97141	0.02799	-1.95510
N	2.13041	0.01134	1.10556
B	-1.96479	0.96482	0.02069
B	-1.95838	-0.95038	0.00354
N	-2.13219	0.01654	-1.10699
N	-1.11391	-2.11378	-0.01213
N	-2.11739	-0.00321	1.13333
N	-1.12820	2.13396	0.02538
B	0.01212	-1.95511	0.93950
B	-0.94555	-0.00662	1.96619
B	-0.00115	1.96625	0.97428
B	0.96969	-0.00003	1.95374
N	0.00934	1.11543	2.13308
N	0.01688	-1.12493	2.11322

N	1.67679	0.01199	3.87341
H	2.66595	-0.16705	3.74727
H	1.61030	0.98460	4.14955
H	1.40664	-0.52137	4.69137

$Ni@b_{64}$-NH_3

B	0.93948	0.00891	-2.00369
N	2.12629	0.02073	-1.19352
N	-0.03588	1.12121	-2.12287
N	-0.01007	-1.12473	-2.14313
B	0.01670	-1.94792	-0.96557
B	0.05268	1.99422	-0.96166
B	1.92222	-0.86038	-0.02909
B	1.96939	1.07196	-0.15611
N	1.36787	2.35345	-0.39365
N	1.14585	-2.06872	-0.00528
B	-0.96693	-0.00777	-1.95641
N	2.00060	0.17530	1.03720
B	-1.92266	0.90926	0.01881
B	-1.96494	-0.98461	0.00375
N	-2.12545	-0.01995	-1.10919
N	-1.09523	-2.13070	0.00097
N	-2.15182	-0.03927	1.13107
N	-1.03193	2.04213	0.05481
B	0.02744	-1.92574	0.94735
B	-1.01074	-0.02750	2.00574
B	-0.21551	2.20331	1.28340
B	0.87177	0.12636	1.93683
N	-0.15844	1.14606	2.27042
N	0.01445	-1.08057	2.10593
Ni	1.18055	3.32012	1.04262
H	1.60149	5.73167	0.44643
H	1.60149	5.99817	2.03763
H	2.91869	5.40557	1.31893
N	1.90769	5.34117	1.32983

$Ni@b_{66}$-NH_3

B	0.93183	0.03456	-1.97911
N	2.10182	0.03131	-1.13743
N	-0.02176	1.15472	-2.12349
N	-0.01398	-1.08947	-2.14314
B	-0.00200	-1.93420	-0.97664
B	-0.01558	1.97901	-0.94240
B	1.96534	-0.93953	-0.03361
B	1.95853	0.98164	-0.01673
N	1.11421	2.13312	0.01134
N	1.12889	-2.09705	-0.02564
B	-0.97674	0.02784	-1.96518
N	2.17251	0.01196	1.09880
B	-1.96899	0.96212	0.01045

B	-1.96237	-0.94765	-0.00617
N	-2.13707	0.01645	-1.12154
N	-1.11083	-2.10658	-0.01820
N	-2.12578	-0.00308	1.12114
N	-1.12555	2.12700	0.01873
B	0.00973	-1.93528	0.94492
B	-0.94163	-0.00620	1.94845
B	-0.00376	1.94679	0.97884
B	1.01238	-0.00113	2.13867
N	-0.00618	1.11664	2.13280
N	0.00162	-1.12557	2.11327
Ni	2.74239	-0.00136	2.96916
N	4.49807	-0.05495	5.20381
H	5.50907	0.00955	5.19291
H	4.19187	0.33555	4.32041
H	4.19187	0.60215	5.91161
